# The Overexpression of Collagen Receptor DDR1 is Associated With Chromosome Instability and Aneuploidy in Diffuse Large B‐Cell Lymphoma

**DOI:** 10.1111/jcmm.70318

**Published:** 2025-05-22

**Authors:** Sandra Margielewska‐Davies, Matthew Pugh, Eszter Nagy, Ciara I. Leahy, Maha Ibrahim, Eanna Fennell, Aisling Ross, Jan Bouchal, Lauren Lupino, Matthew Care, Reuben Tooze, Gary Reynolds, Zbigniew Rudzki, Wenbin Wei, William Simmons, Vikki Rand, Kelly Hunter, John J. Reynolds, Grant S. Stewart, Katerina Bouchalova, Iona J. Douglas, Katerina Vrzalikova, Paul G. Murray

**Affiliations:** ^1^ Institute of Immunology & Immunotherapy University of Birmingham Birmingham UK; ^2^ Institute of Cancer and Genomic Sciences University of Birmingham Birmingham UK; ^3^ Limerick Digital Cancer Research Centre, Bernal Institute and Health Research Institute and School of Medicine University of Limerick Limerick Ireland; ^4^ South Egypt Cancer Institute Assiut University Assiut Egypt; ^5^ Department of Clinical and Molecular Pathology, Institute of Molecular and Translational Medicine, Faculty of Medicine and Dentistry Palacky University and University Hospital Olomouc Olomouc Czech Republic; ^6^ Experimental Haematology, Leeds Institute of Cancer and Pathology University of Leeds Leeds UK; ^7^ Department of Histopathology Birmingham Heartlands Hospital, University Hospitals Birmingham NHS Foundation Trust UK; ^8^ The Palatine Centre Durham University Durham UK; ^9^ National Horizons Centre Teesside University Darlington UK; ^10^ School of Health and Life Sciences Teesside University Middlesbrough UK; ^11^ School of Biosciences Aston University Birmingham UK; ^12^ Department of Pediatrics, Faculty of Medicine and Dentistry Palacky University and University Hospital Olomouc Olomouc Czech Republic; ^13^ West Midlands Regional Genetics Laboratory Birmingham Women's NHS Foundation Trust Birmingham UK; ^14^ Royal College of Surgeons in Ireland Medical University of Bahrain Manama Bahrain

**Keywords:** aneuploidy, CENPE, chromosome instability, collagen, DDR1, DLBCL, mitotic spindle, *TP53*

## Abstract

Although chronic inflammation is implicated in the pathogenesis of diffuse large B‐cell lymphoma (DLBCL), the mechanisms responsible are unknown. We demonstrate that the overexpression of the collagen receptor, DDR1, correlates with reduced expression of spindle checkpoint genes, with three transcriptional signatures of aneuploidy and with a higher frequency of copy number alterations, pointing to a potential role for DDR1 in the acquisition of aneuploidy in DLBCL. In support of this, we found that collagen treatment of primary germinal centre B cells transduced with DDR1, not only partially recapitulated the aberrant transcriptional programme of DLBCL but also downregulated the expression of CENPE, a mitotic spindle that has a crucial role in preventing chromosome mis‐segregation. CENPE expression was also downregulated following DDR1 activation in two B‐cell lymphoma lines and was lost in most DDR1‐expressing primary tumours. Crucially, the inhibition of CENPE and the overexpression of a constitutively activated *DDR1* were able to induce aneuploidy in vitro. Our findings identify a novel mechanistic link between DDR1 signalling and chromosome instability in B cells and provide novel insights into factors driving aneuploidy in DLBCL.

## 1 Introduction

Diffuse large B‐cell lymphoma (DLBCL) is the most prevalent form of B‐cell lymphoma, accounting for 30%–40% of newly diagnosed cases. Despite intense R‐CHOP immunochemotherapy, up to one‐third of patients have disease that is refractory, or which will relapse. Although patients with relapsed/refractory DLBCL are offered intensive salvage chemotherapy and autologous transplantation, this is only successful in around 20% of cases [1].

The Cell of Origin Classification recognises at least two major subdivisions of DLBCL which arise from distinct stages of B‐cell differentiation; the activated B‐cell (ABC) type, derived from late germinal centre (GC) or post‐GC B cells, and the germinal centre B (GCB) type, originating from an earlier, probable GC, stage [2]. These two major forms of DLBCL are distinguished based on gene expression differences and underlying genetics and oncogenic signalling pathways [2]. Patients with the ABC subtype have inferior survival. [2] Recently, several groups have identified different molecular entities, including an ABC/GCB‐independent group displaying bi‐allelic inactivation of *TP53*, and genomic instability with a high frequency of somatic copy number alterations (SCNAs) [3, 4].

Chromosome instability (CIN) may underlie the development of genetic aberrations found in DLBCL, including SCNA [5], and is associated with adverse outcomes [6, 7]. For example, a twofold increase in the frequency of chromosome mis‐segregation was associated with a 24% decrease in overall survival and a 48% decrease in relapse‐free survival. [6] Patients with evidence of chromosome mis‐segregation were also more likely to present with higher tumour stage and higher international prognostic index (IPI) scores [6]. Mechanisms underpinning the development of CIN in DLBCL might involve the spindle assembly checkpoint (SAC), a multiprotein signalling cascade which detects the presence of misoriented or detached kinetochores and arrests cells in metaphase until all sister chromatid pairs are bi‐oriented ensuring their equal separation during cell division [8]. The SAC comprises several proteins located at kinetochores, including the mitotic arrest‐deficient (MAD) proteins (MAD1, MAD2 and MAD3), budding uninhibited by benzimidazole (BUB) proteins (BUB1, BUB2 and BUB3/BUBR1), monopolar spindle 1 protein (MPS1), ROD‐ZW10‐Zwilch complex and the microtubule motor centromere protein E (CENPE) [9]. In the mouse, germ‐line deletion of SAC genes results in early embryonic lethality, whereas heterozygous knockout of *MAD2* and other SAC genes generates relatively weak tumour phenotypes late in life. Paradoxically, some SAC mutations (e.g., *CENPE* heterozygosity) can be both tumour predisposing and tumour suppressing, depending on cellular context [10]. Of particular interest here is the observation that mice with heterozygous knockout of *MAD2* crossed with a heterozygous knockout of *TP53* showed a substantially increased lymphoma incidence [11], suggesting p53 defects might override the SAC.

Features of the DLBCL tumour microenvironment (TME) have been associated with patient outcomes [12, 13]. For example, the expression of genes encoding collagens and regulators of collagen synthesis are associated with patient outcomes in DLBCL. [13] Collagen receptors implicated in cancer pathogenesis [14, 15], include the receptor tyrosine kinases and discoidin domain receptor (DDR)‐1 and ‐2 [16]. *DDR1* is activated by many collagen types (e.g., I, IV, V, VI and VIII), whereas DDR2 is only activated by fibrillar collagens (e.g., I, III and X) [17]. DDR1 and DDR2 regulate a variety of cellular processes, including proliferation, apoptosis and migration, as well as inflammation, neo‐angiogenesis and metastasis [18, 19, 20]. DDR1 has also recently gained attention as a potential therapeutic target due to its ability to promote therapy resistance in several cancer types [21, 22]. Previously, we showed that *DDR1* is overexpressed in Hodgkin lymphoma and that DDR1 activation by collagen enhanced the survival of B‐cell lymphoma lines [23]. Here, we explore the role of DDR1 in the pathogenesis of DLBCL.

## 2 Materials and Methods

### 2.1 Tissue Samples

FFPE samples of DLBCL were from UHB Trust, Birmingham, UK, and their use was approved by West Midlands and The Black Country committees of the National Research Ethics Service, UK (REC:14/WM/0001). Fresh paediatric tonsils were obtained with informed consent under local ethics committee approval (No. 06/Q2702/50).

### 2.2 CD10 Isolation, Transfection and RNA Sequencing

CD10‐positive GC B cells were purified and transfected as before [24, 25] using control or *DDR1α* containing pIRES2–EGFP vectors (gift, Simon Johnson, University of Nottingham). Consistent with our previous observations, GC B cells maintained a representative GC phenotype after sorting and transfection [26]. Eight hours posttransfection, soluble type‐I collagen was added (Millipore Ltd., Watford, UK) (2 h; 100 μg/mL) and cells were harvested following collagenase (Sigma‐Aldrich, Dorset, UK) treatment (10 min, 250 μg/mL). CD10‐positive, GFP‐positive and Hoechst‐negative cells were enriched by MoFlo sorting, RNA was extracted (RNeasy microkit, QIAGEN Ltd., Manchester, UK) and cDNA was amplified using Ovation RNA‐Seq kit (NuGEN Ltd., Leek, The Netherlands). For RNA‐seq, TruSeq Nano libraries were generated from cDNA and sequenced on HISeq4000 for Illumina paired‐end RNA sequencing (Edinburgh Genomics, Edinburgh, UK, Data S1). To identify whether there was a significant overlap between two gene expression datasets, we used only those genes present on both gene expression platforms by Venny: https://bioinfogp.cnb.csic.es/tools/venny/. A Chi‐square test was used to determine if the number of genes in the overlap between two datasets was significantly higher or lower than expected by chance, and the *p*‐value computed from the Chi‐square value using 1 degree of freedom at https://www.socscistatistics.com/pvalues/chidistribution.aspx.

### 2.3 Transfection of Cell Lines

DG75 and BJAB B‐cell lymphoma lines (DSMZ, Braunschweig, Germany) were cultivated in RPMI 1640 (Gibco; Life Technologies Ltd., Paisley, UK) culture media, with 10% FCS and 1% penicillin–streptomycin (Gibco). Prior to experiments, cells were tested for the presence of mycoplasma (MycoAlert Mycoplasma Detection Kit; Lonza, Slough, UK). DG75 and BJAB cells were nucleofected using program R013 (Lonza Biologicals, Slough, UK) with plasmids for *wtDDR1* (as above) or pCDH‐*mDIV‐DDR1* and pCDH‐*DIV‐DDR1* (gift, Dr. Gaoxiang Ge, Shanghai Institutes for Biological Sciences).

### 2.4 Inhibitor Treatment

Cells were ‘serum starved’ for 2 h, followed by incubation with 50 nM CENPE inhibitor (GSK923295; Cayman Chemical, Michigan, USA) for 4 h and 2 μM MPS1 inhibitor (AZ3146; ApexBio, Boston, USA) for 2 h. After 72 h, metaphase spreads were prepared.

### 2.5 Chromosome Counts

Metaphase spreads were prepared by incubation in colcemide (10 μg/mL; Sigma‐Aldrich) for 3 h followed by wash in hypotonic buffer and fixation in 25% acetic acid in ethanol. Cells were dropped onto a glass slide and G‐banded. Metaphases were captured on a Metasystems Metafer slide scanning system. Chromosome counting was performed in triplicate in ImageJ, counting only nonoverlapping chromosomes. To quantify aneuploidy, only hyperploid metaphases were considered. While the typical karyotype of DG75 cells is 46XY; *t* (8:14), a conservative threshold of 48 was used to define hyperploidy and avoid overcalling [27]. Pearson Chi‐square test and ‘column proportion test’ with Bonferroni correction were performed. To assess interrater reliability, 61 of the metaphases were counted by three investigators and interclass correlation coefficient and 95% confidence intervals were calculated based on a mean rating (*k* = 3), absolute agreement and a two‐way mixed‐effects model.

### 2.6 Immunostaining/Blotting

Immunostaining was as described previously [28] using antibodies in Table S1A. For CENPE and DDR1 staining, tumour samples were recorded as positive if ≥ 25% of tumour cells were positive for each marker. For COLVI staining, samples were recorded as positive if ≥ 25% of tumour cells were intimately associated with collagen fibres. Images were obtained using Nikon Eclipse E400 microscope (RT; objectives ×20, ×40; NA 0.40, 0.65) and Nikon DS‐Fi‐1 camera. For Ki67 staining, positive tumour cells were recorded as a percentage of total numbers of morphologically evaluable tumour cells in 10 hpf. For cell lines, digital semiautomated quantitative scoring was performed using Vectra scanner and Inform software (Data S1; Table S1B). Proteins were also detected by SDS‐polyacrylamide gel electrophoresis and standard immunoblotting detected by chemiluminescence (ECL; GE Healthcare, Chalfont St Giles, UK) on a ChemiDoc MP (Bio‐Rad, Watford, UK).

### 2.7 RT‐qPCR

RNA was isolated from cell lines with RNeasy Mini kit including genomic DNA removal using RNase‐Free DNase Set (Qiagen Ltd), and complementary DNA (cDNA) was synthesised with qScript cDNA SuperMix (QuantaBio, Beverly, MA, USA). All gene transcripts were quantified by qPCR with commercial gene expression assays using the ABI Prism 7700 sequence detection system (Applied Biosystems, Thermo Fisher Scientific, Paisley, UK). Target gene values were normalised against endogenous control, and relative gene expression was calculated by delta (ΔΔ) C_t_ method. The normalised values are shown relative to the reference sample that was set to a relative quantity value of 1. All reactions were run in triplicate. Details of Taqman primer/probe qPCR assays are shown in the Table S1C.

## 3 Results

### 3.1 Overexpression of DDR1 in DLBCL

**FIGURE 1:**
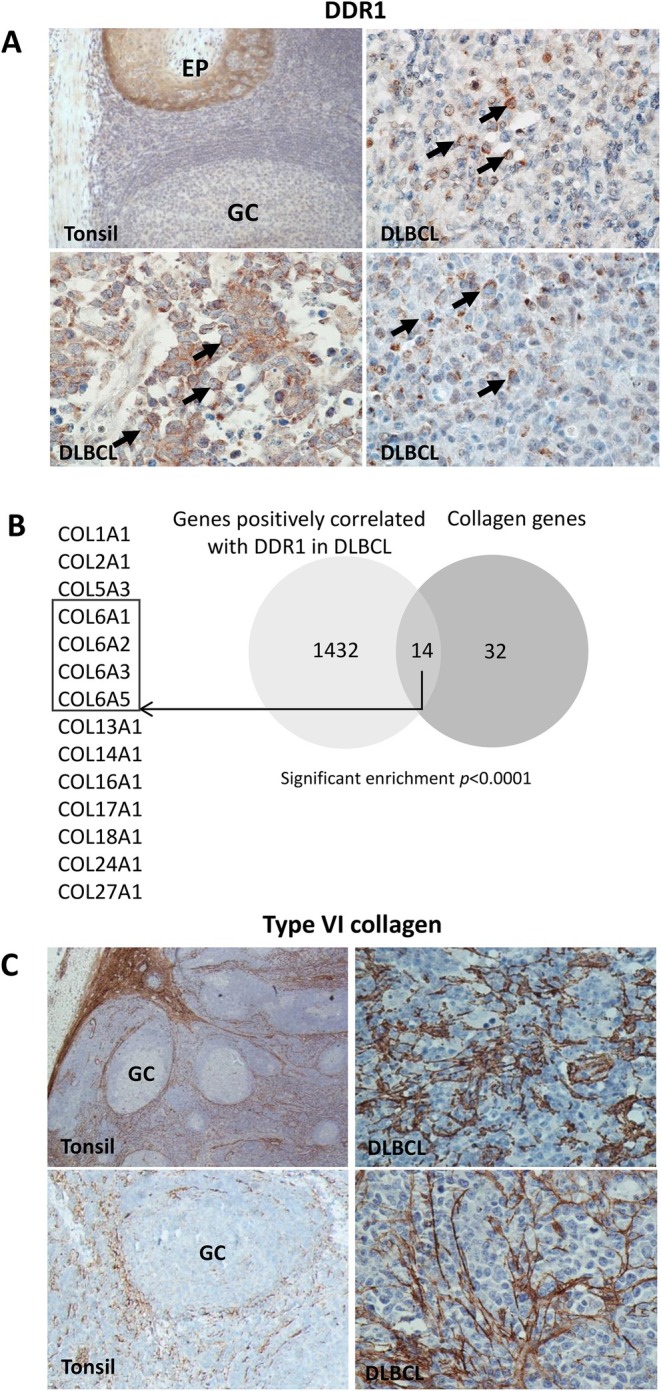
Overexpression of *DDR1* in diffuse large B‐cell lymphoma. (A) Immunohistochemistry showing representative examples of DDR1 staining in normal tonsil (upper left panel; original magnification ×200); germinal centre (GC) B cells did not stain for DDR1, in contrast to positive staining in normal tonsillar epithelium (EP). Remaining panels show tumour cell expression of DDR1 protein (arrows) in three representative cases of DLBCL. Original magnification ×400. (B) Meta‐analysis of 11 DLBCL gene expression datasets comprising over 2000 cases of DLBCL revealed that collagen genes were significantly enriched among genes positively correlated with *DDR1* in primary DLBCL. Collagen genes positively correlated with *DDR1* included *COL6A1*, *COL6A2*, *COL6A3* and *COL6A5* (box). (C) Representative examples of staining for Type VI collagen in tonsil (left panels; original magnification ×200) and primary DLBCL (right panels; original magnification ×400). Type VI collagen was mostly absent from normal germinal centres (GCs), whereas DDR1‐expressing primary DLBCL displayed prominent Type VI collagen deposition surrounding tumour cells.

We first investigated the expression of DDR1 in primary DLBCL using immunohistochemistry and a monoclonal antibody that we showed was DDR1 specific (Figure S1A,B). Normal GC B cells did not express DDR1. However, DDR1 protein was detected in tumour cells in 38/90 cases (Figure 1A), confirmed by costaining for CD20 and DDR1 (Figure S1C). We stained all cases for BCL6, CD10 and IRF4, and using the Hans algorithm, defined each case as either GCB or non‐GCB type [28, 29]. A total of 13/41 GCB and 25/48 non‐GCB‐DLBCL were DDR1‐positive and one was unclassifiable (Table S2). We conclude that DDR1 is overexpressed in a subset of DLBCL, including both GCB‐ and non‐GCB‐DLBCL.

Re‐analysis of datasets reporting global gene expression in DLBCL and normal GC B cells revealed that when compared to primary GC B cells, DDR1 mRNA was significantly overexpressed in a subset of DLBCL, including cases of both ABC and GC type (data not shown; *p* = 0.020) [30, 31, 32]. Comparison of DDR1 expression in a series of DLBCL reported by Morin et al. [30, 31] and Lenz et al. [13] revealed that DDR1 expression was significantly higher in GCB‐type tumours compared to ABC‐type DLBCL in both datasets (Lenz et al., *p* ≤ 0.0001; Morin et al., *p* = 0.017).

### 3.2 DDR1‐Expressing DLBCL are Enriched for Collagens

We next wanted to determine if there was any relationship between DDR1 and the expression of its collagen ligands in DLBCL. We first confirmed that DDR1 mRNA levels could be used as a surrogate of DDR1 protein expression using a panel of DLBCL lines. Figure S2 shows that the levels of DDR1 mRNA matched with levels of the DDR1 protein in these cell lines. We then performed a meta‐analysis of 11 DLBCL mRNA expression datasets comprising > 2000 cases [33]. For each data set, the variance for each gene was used to order them by patient sample, and Spearman's rank correlations compared to that of DDR1 were calculated from the top 80% of the genes. The correlation matrices and *p*‐values were merged across all datasets using the median values. A *DDR1*‐correlated gene set was created by taking all genes present in at least six datasets with a median *p* < 0.05. A total of 1446 genes were positively correlated, and 1295 genes were negatively correlated, with *DDR1* expression. Collagen genes were significantly enriched among genes positively correlated with *DDR1* mRNA (odds ratio (OR) = 5.69; *p* < 0.0001; Table S3; Figure 1B), including *COL6A1*, *COL6A2*, *COL6A3* and *COL6A5*, encoding Type VI collagen subunits. By immunohistochemistry, normal GCs lacked Type VI collagen, whereas both DDR1‐positive and DDR1‐negative DLBCL showed prominent deposition of Type VI collagen surrounding tumour cells (Table S2; Figures 1C and S1D,E). Although by quantitative analysis, DDR1‐positive cases had a higher density of Type VI collagen protein expression (Figure S1F), this difference was not statistically significant.

### 3.3 Genes Negatively Correlated With DDR1 are Enriched for Mitotic Spindle Genes

**FIGURE 2:**
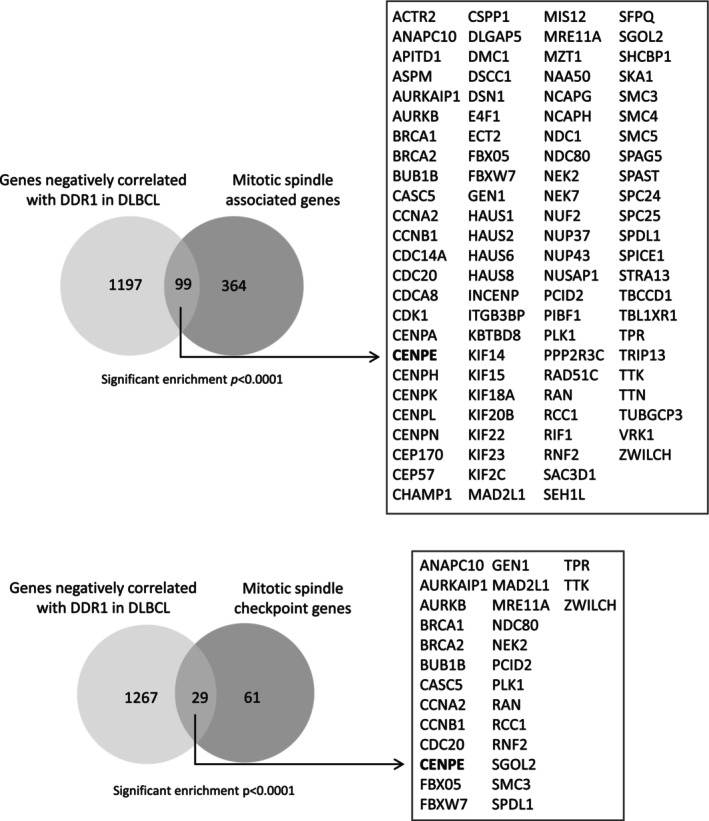
Genes negatively correlated with *DDR1* expression in DLBCL are enriched for mitotic spindle‐associated genes. Significant enrichment of ‘mitotic spindle associated’ genes (top panel) and ‘mitotic spindle checkpoint’ genes (bottom panel) among genes negatively correlated with *DDR1* expression in DLBCL (total number of genes on both platforms = 20,121).

A gene ontology (GO) analysis revealed that genes positively correlated with *DDR1* expression in DLBCL were enriched for GO terms including ‘collagen catabolic process’, ‘collagen metabolic process’ and ‘wound healing’ as well as ‘regulation of apoptosis’ and ‘cell migration’, reflecting known DDR1 functions (Figure S3A). On the other hand, genes negatively correlated with *DDR1* were enriched for GO terms that included ‘mitotic spindle organization’ and ‘mitotic sister chromatid segregation’ (Figure S3B). To further explore the possibility that genes with mitotic spindle functions might be downregulated in *DDR1*‐expressing DLBCL, we utilised a list of 513 ‘mitotic spindle associated’ genes, including the subset of genes associated with the mitotic spindle checkpoint (GO:0031577) (Table S4A,B). We found that ‘mitotic spindle associated’ genes, including those encoding the mitotic spindle checkpoint, were enriched among genes negatively correlated with *DDR1* expression in DLBCL (OR = 3.67; *p* < 0.0001; and OR = 7.03; *p* < 0.0001, respectively; Figure 2). We conclude that *DDR1*‐expressing DLBCL has reduced expression of mitotic spindle genes.

### 3.4 DDR1 Expression Correlates With Transcriptional Signatures of Aneuploidy

**FIGURE 3:**
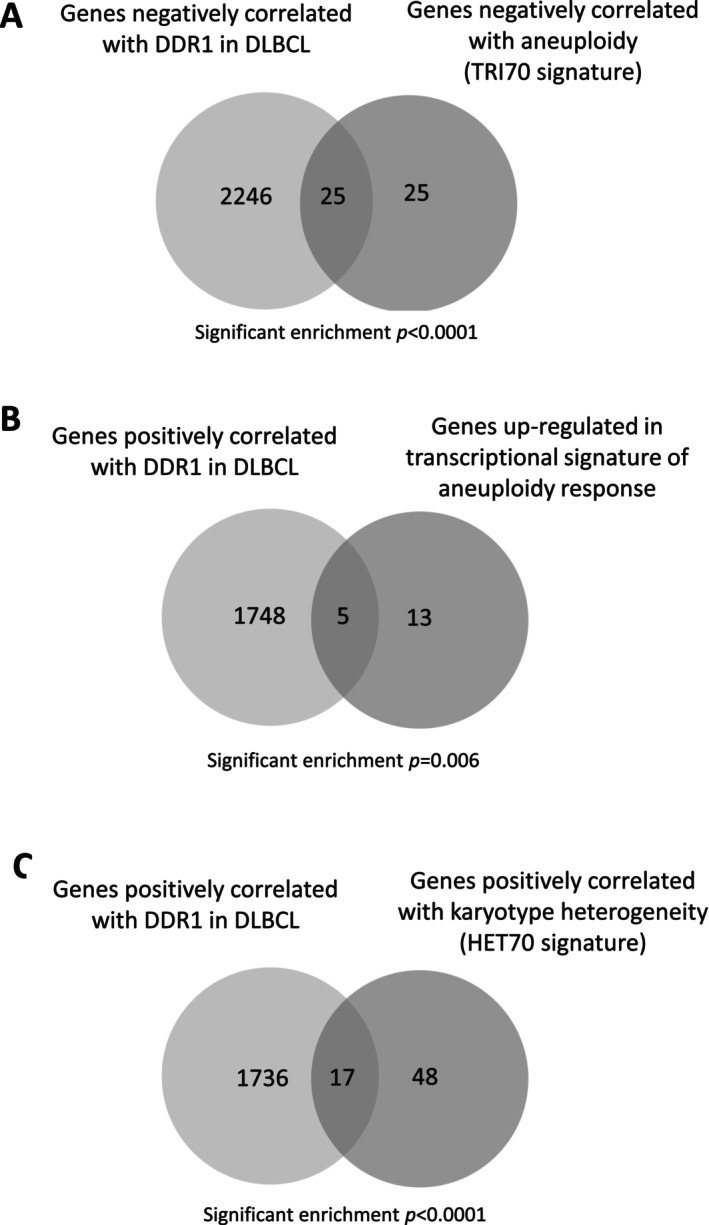
*DDR1* expression correlates with an aneuploidy‐associated transcriptional programme in primary DLBCL. (A) Genes negatively correlated with *DDR1* expression in DLBCL were enriched among genes negatively correlated with aneuploidy in the TRI70 signature. (B) Genes positively correlated with *DDR1* in DLBCL were also enriched among genes upregulated in aneuploid versus diploid cells. (C) Genes positively correlated with *DDR1* expression in DLBCL were enriched among those displaying the strongest positive correlation with karyotype heterogeneity in the NCI60 panel of cell lines (HET70 signature). For all comparisons, the total number of genes on both platforms = 20,119.

We next explored if *DDR1* expression was associated with aneuploidy‐associated transcription. First, we used the TRI70 gene set, which contains 50 genes displaying the strongest absolute negative correlation with aneuploidy in trisomic MEFs [34]. Genes negatively correlated with aneuploidy in TRI70 were enriched for genes negatively correlated with *DDR1* expression in DLBCL (*p* < 0.0001; OR = 7.92; Figure 3A). We used a second transcriptional signature derived from multiple aneuploid versus diploid datasets [35]; genes upregulated in this core aneuploidy signature were significantly enriched for genes positively correlated with *DDR1* expression (*p* = 0.0061; OR = 4.04; Figure 3B). To exclude the possibility that the enrichment of aneuploidy signatures among *DDR1*‐correlated genes was simply a reflection of the reduced expression of proliferation‐associated genes, we used the HET70 aneuploidy signature, consisting of genes with the strongest positive correlation with karyotype heterogeneity in the NCI60 cell line panel and derived specifically to distinguish aneuploidy and proliferation‐associated transcriptional programmes [34]. We found that HET70 genes were also significantly enriched among genes positively correlated with *DDR1* expression (*p* < 0.0001; OR = 3.74; Figure 3C). We conclude that *DDR1* expression correlates with an aneuploidy‐associated transcriptional programme in primary DLBCL.

### 3.5 DDR1 Expression Is Associated With a Higher Frequency of SCNAs in *TP53* ‐Mutant DLBCL

**FIGURE 4:**
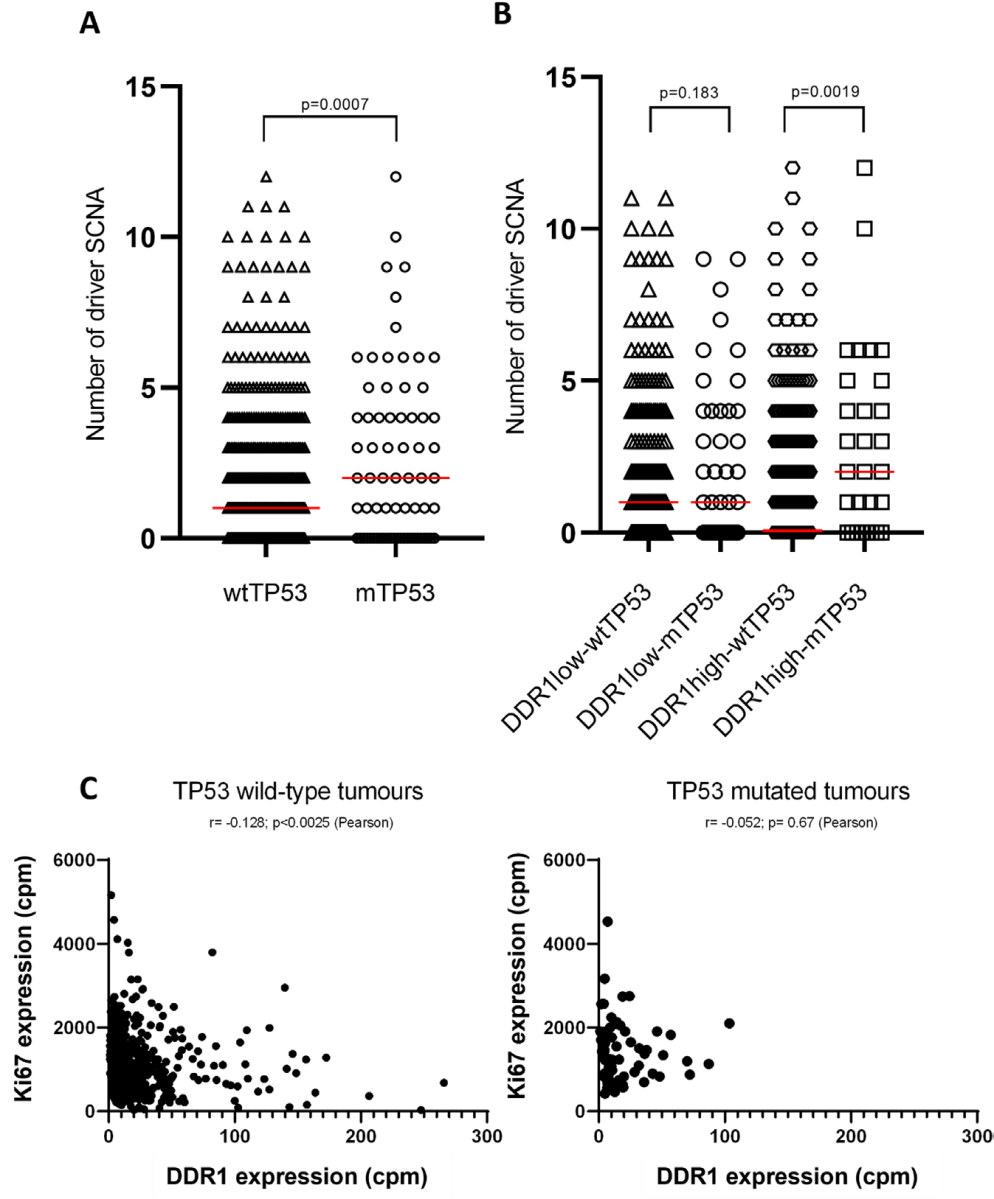
Higher frequency of SCNA defined as ‘driver genes’ in *TP53*‐mutant DLBCL overexpressing DDR1. (A) The number of genes with SCNA defined as ‘driver genes’ in Chapuy et al. is significantly higher in *TP53‐*mutant versus *TP53* wild‐type DLBCL. (B) Analysis separated into *DDR1*‐low and *DDR1*‐high subgroups (split by median) showing a significantly increased frequency of driver genes with SCNA in *TP53‐*mutant versus *TP53* wild‐type tumours, but only in the *DDR1*‐high group. (C) *TP53* wild‐type tumours show a significant negative correlation between *DDR1* and *Ki67* expression; an effect not observed in *TP53*‐mutant tumours.

In mouse models heterozygous for genetic knockout of the mitotic spindle checkpoint, reduced *TP53* function resulted in a significant increase in the risk of lymphoma, suggesting that *TP53* loss of function might be required for the propagation of CIN [11]. Moreover, in a recent study, a subset of DLBCL with a high frequency of SCNAs was shown to also be enriched for *TP53* mutations [3]. To explore the relationship between *DDR1* expression, *TP53* status and the frequency of SCNA, we reanalysed the cohort of 624 DLBCL cases reported by Reddy et al. [36], which include data on copy number for 140 driver genes, *TP53* mutation status and global gene expression. We first confirmed in this dataset that SCNAs are higher in tumours with *TP53* mutations compared to those with wild‐type *TP53* (*p* = 0.0007; Figure 4A). However, when we split the tumours (at the median) into those expressing higher or lower *DDR1* expression, we found that among the group of tumours with higher *DDR1* expression, those harbouring a *TP53* mutation had a significantly higher copy number score than the group with no *TP53* mutation (Mann–Whitney *U* test, *p* = 0.0019, Figure 4B). However, in tumours with lower *DDR1* expression, there was no significant difference in copy number between the cases harbouring a mutant or a wild‐type *TP53* (*p* = 0.18, Figure 4B). These data suggest that the differences in SCNA frequency between *TP53*‐mutant and *TP53* wild‐type tumours shown in Figure 4A are primarily driven by differences in SCNA frequency among tumours expressing higher levels of *DDR1*. While, as expected, *Ki67* expression was significantly higher in *TP53*‐mutant tumours compared to those tumours with a wild‐type *TP53* gene (*p* = 0.0024, data not shown), we noted that *DDR1* expression was negatively correlated with *Ki67* expression in *TP53* wild‐type tumours, but not in *TP53*‐mutant tumours (Figure 4C). We also explored the relationship between *DDR1* expression and SCNA frequency in a second dataset reported by Chapuy et al. [3] As expected, the number of driver SCNAs was higher in *TP53*‐mutant cases (not shown). Comparing *DDR1* expression across the different genetic subtypes defined by Chapuy et al. [3] revealed higher expression in the C2 subtype, defined by a high frequency of bi‐allelic inactivation of *TP53* and genomic instability compared with C1, C3 and C4 subtypes, although these differences were not significant (*p* = 0.07, *p* = 0.13, *p* = 0.06, respectively; Figure S4).

### 3.6 Activated DDR1 Downregulates Expression of the Mitotic Kinesin, CENPE

**FIGURE 5:**
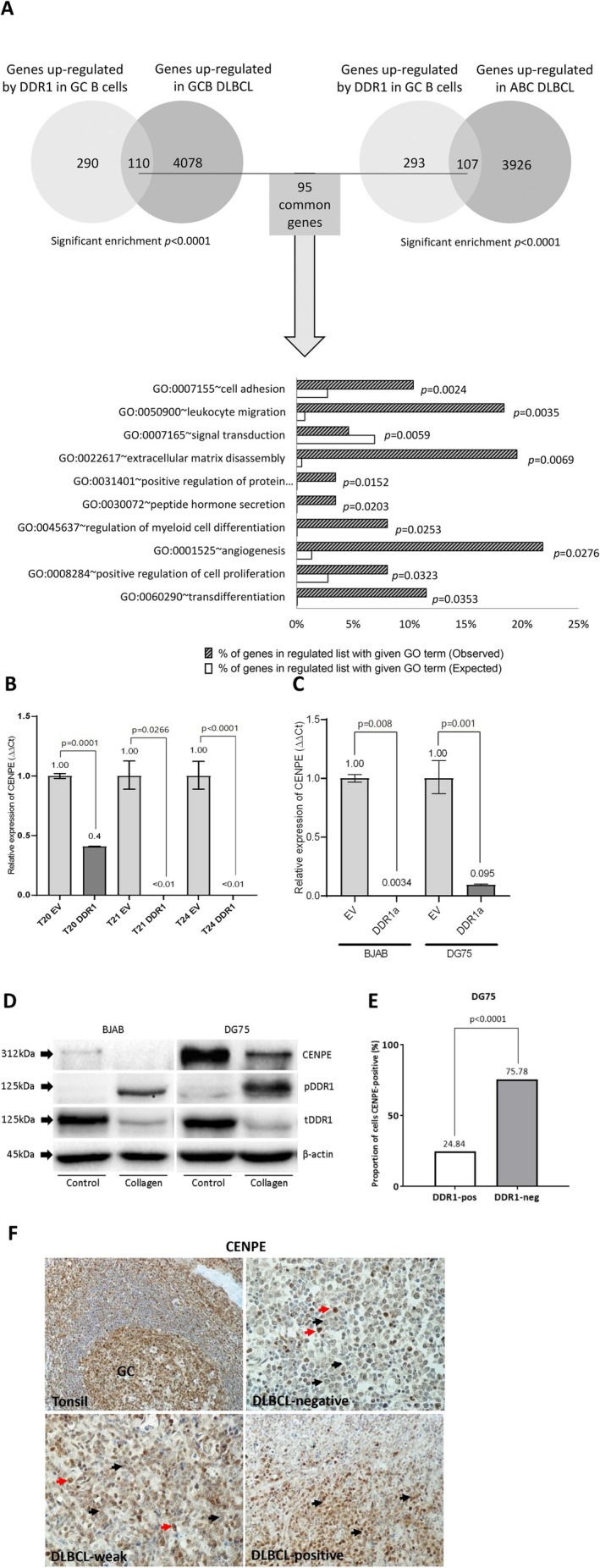
Activated *DDR1* downregulates expression of the mitotic kinesin, *CENPE*. (A) Upper panels show the significant overlap between genes upregulated by DDR1 in normal GC B cells and those upregulated in either GCB‐ or ABC‐DLBCL compared with normal GC B cells (total genes on both platforms = 26,438). The lower panel shows GO analysis of genes upregulated by DDR1 in normal GC B cells and also upregulated in both GCB and ABC‐DLBCL. Open bars indicate the percentage of genes present in the GO term that would be expected to be observed in the regulated gene list by chance. Shaded bars indicate the percentage of genes in the regulated list with a given GO term that were actually observed. (B) RT‐qPCR confirming that collagen treatment of DDR1‐expressing GC B cells significantly reduced the mRNA levels of *CENPE*. Shown are triplicate data on tonsillar GC B cells isolated from three different donors (T20, T21 and T42). (C) Compared to empty vector (EV) control, the addition of collagen significantly reduced *CENPE* mRNA expression in DDR1‐expressing B‐cell lines, BJAB (maximally after 6 h of stimulation) and DG75 (maximally after 2 h of stimulation). Data are representative of three separate biological replicates. (D) Activation of an ectopically expressed DDR1 reduced CENPE protein expression in both BJAB and DG75 cells. An antibody specific for DDR1 phosphorylated on Tyr792 was used to confirm activation of DDR1. As expected, the activation of DDR1 also reduced total DDR1 levels. β‐Actin was used as a loading control. Data shown are representative of three separate biological replicates. (E) A constitutively activated *DDR1* construct also downregulated CENPE protein expression. Shown here are the mean fluorescence intensities for DDR1 and CENPE in DG75 cells transfected with the constitutively active *DDR1* gene. Cells expressing DDR1 were significantly more likely to lack CENPE protein expression than were untransfected cells in the same population (*p* < 0.0001). (F) Immunohistochemistry showing the downregulation of CENPE protein in DLBCL tumour cells. Top left panel shows strong expression of CENPE protein in a normal germinal centre (GC) of tonsil. Original magnification ×200. Remaining panels show examples of staining in a case in which CENPE was not detected in tumour cells (top right), a case with weak expression in tumour cells (bottom left) and a case showing strong staining (bottom right). Original magnification ×400. Black arrows show tumour cells. Red arrows indicate nonmalignant cells that are positive for CENPE.

Having shown that *DDR1* expression is associated with a higher frequency of SCNA in DLBCL, we next wanted to explore if this was due to the transcriptional regulation of key mitotic spindle genes by DDR1. We transfected normal primary GC B cells with *DDR1* and empty control vector using an approach we have described previously [24, 25], after which we confirmed the expression of DDR1 (Figure S5A). RNA sequencing was performed after treating transfected cells with collagen. Collagen treatment of DDR1‐expressing GC B cells was followed by the upregulation of 400 unique genes (Table S5A) and the downregulation of 260 unique genes (Table S5B). We found a striking overlap between genes upregulated by DDR1 in normal GC B cells and genes that were upregulated in both ABC (OR = 2.04; *p* < 0.00001) and GCB subtypes of DLBCL (OR = 2.03; *p* < 0.00001; Figure 5A). GO analysis of the 95 genes upregulated by DDR1 expression in GC B cells and differentially expressed in both GCB‐ and ABC‐DLBCL compared to normal GC B cells revealed the enrichment of genes with functions in ‘cell adhesion’, ‘leukocyte migration’, ‘angiogenesis’ and ‘positive regulation of cell proliferation’, reflecting known functions of DDR1 (Figure 5A). We validated the differential expression of a subset of DDR1 targets in both DDR1‐expressing GC B cells and B‐cell lymphoma lines using RT‐qPCR (Figure S5B,C). Given that we had previously observed a striking overlap between genes negatively correlated by DDR1 and genes with a function in the mitotic spindle checkpoint, we focused our attention on *CENPE*, which was downregulated by collagen treatment of DDR1‐expressing primary GC B cells (fold change = −2.31; *p* < 0.05). CENPE was of particular interest because it has been shown to be essential for proper chromosome segregation. Thus, the decreased expression of CENPE can induce chromosome mis‐segregation and in some cases has been shown to be sufficient to induce aneuploidy [37, 38]. CENPE is also one of a number of cell‐cycle–related genes known to be upregulated during the G_2_‐M phase [39]. We used RT‐qPCR to confirm the decreased expression of *CENPE* mRNA in collagen‐treated DDR1‐expressing GC B cells and B‐cell lymphoma lines (Figure 5B,C). We validated an antibody specific for CENPE protein (Figure S6A) and used this to show that CENPE protein levels were decreased by collagen treatment of the DDR1‐expressing B‐cell lymphoma lines (Figure 5D) and by expression of a constitutively activated *DDR1* construct (Figure 5E). Immunohistochemistry showed that CENPE protein levels were reduced in the tumour cells of DDR1‐positive cases of DLBCL (Figures 5F and S6B; Table S2). CENPE expression status did not correlate with the proliferative index of tumours (unpaired *t*‐test, *p* = 0.13). Our data show that DDR1 activation downregulates the expression of CENPE, suggesting that DDR1 might be directly involved in the development of CIN in DLBCL.

### 3.7 CENPE Inhibition Induces Chromosome Mis‐Segregation in Lymphoma Cell Lines Harbouring Mutant *TP53*

**FIGURE 6:**
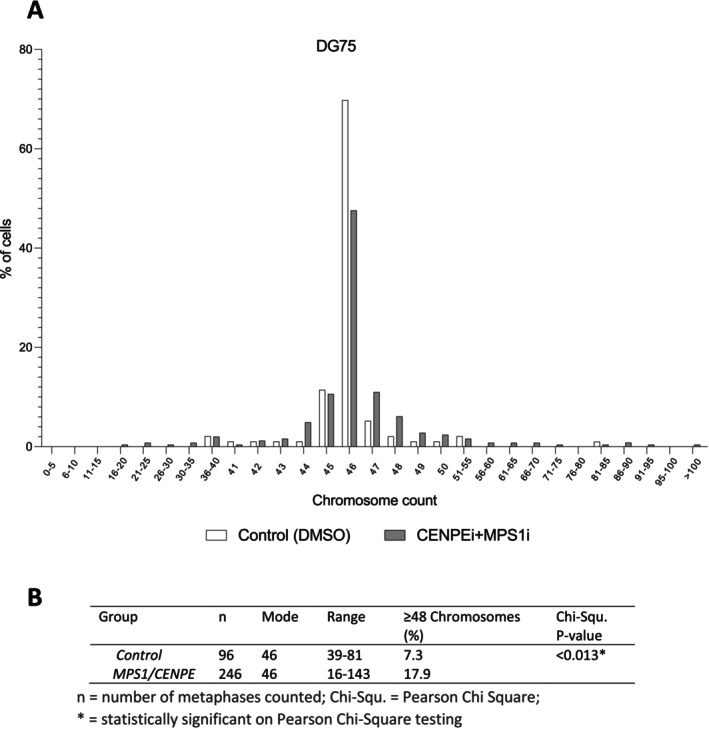
Inhibition of CENPE and MPS1 induces aneuploidy in DG75 cells. (A) DG75 cells treated with 50 nM CENPE inhibitor (GSK923295), followed by 2 μM MPS1 inhibitor (AZ3146) treatment showed increase in aneuploidy population after 72 h, in comparison to nontreated control. (B) Table shows number of counted cells, range of chromosomes and statistical analysis of the chromosome counts.

Next, we tested if CENPE inhibition could induce aneuploidy in B cells. We treated the *TP53*‐mutant and karyotypically stable B‐cell line, DG75, with GSK923295, an allosteric inhibitor of CENPE that prevents ATP hydrolysis, stabilising the enzyme in a conformation that has increased affinity for microtubule binding [38, 40]. While the majority of parental DG75 cells treated with GSK923295 assembled bipolar spindles and aligned most of their chromosomes, in some cells, chromosomes clustered near the spindle poles. However, these cells did not become aneuploid consistent with prior reports (not shown) [38]. In contrast, treatment of GSK923295‐exposed cells with the MPS1 kinase inhibitor, AZ3146, which overrides the SAC allowing anaphase progression, leads to the emergence of aneuploid cells (Figure 6).

### 3.8 Constitutive Activation of DDR1 Induces Aneuploidy

**FIGURE 7:**
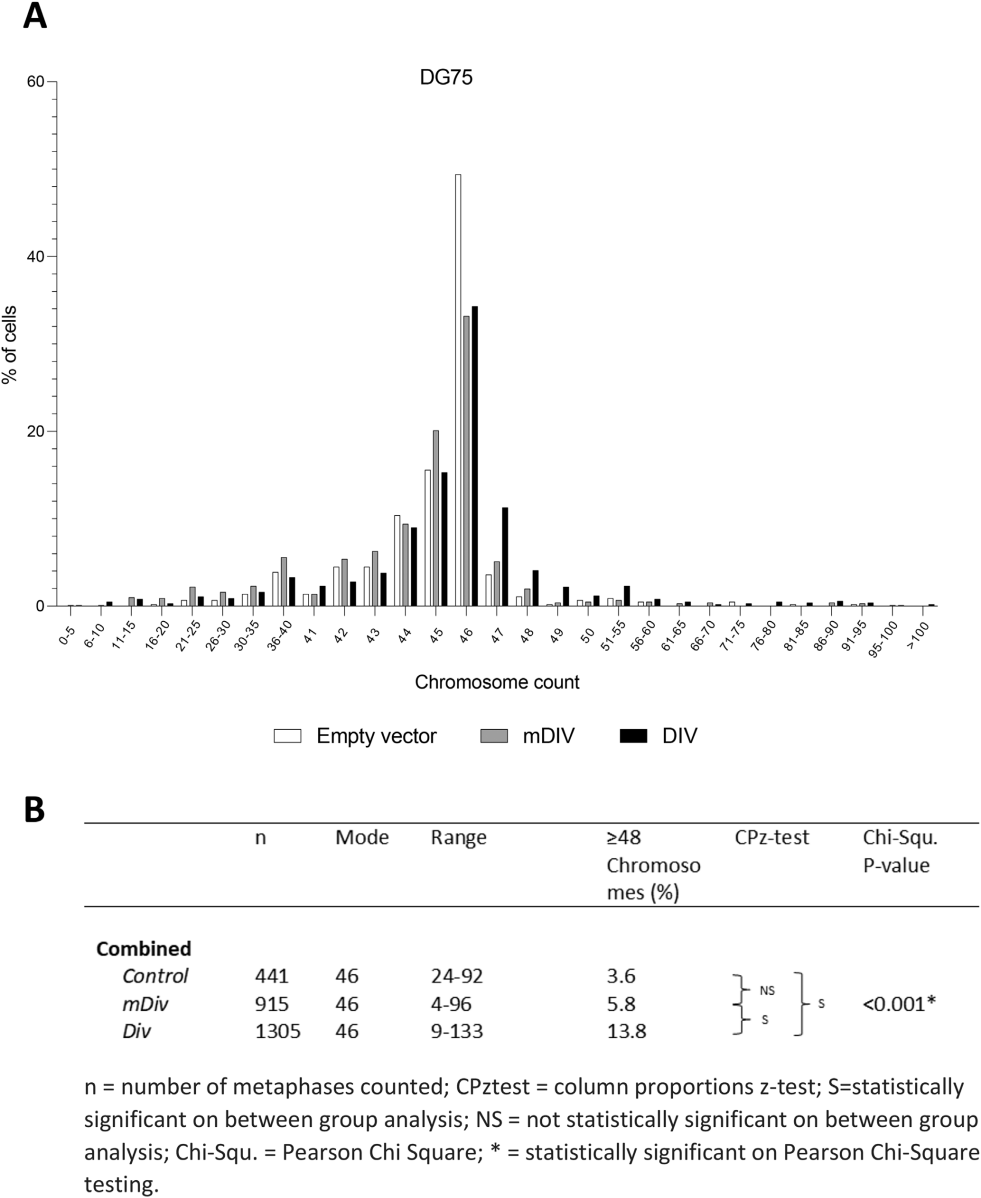
Activation of DDR1 induces chromosomal instability in the *TP53*‐mutant cell line DG75. (A) DG75 cells transfected with constitutively active *DDR1* (*DIV*), mutant receptor (*mDIV*) or untransfected cells. In comparison to controls at 72 h, *DIV*‐transfected cells showed an increase in the number of hyperploid cells. (B) Table showing statistical analysis of chromosome counts for three replicates combined.

We next investigated if DDR1 could directly induce CIN. We transduced DG75 cells with a chimeric receptor (*DIV‐DDR1*) constructed by replacing the extracellular ligand binding discoidin domain of DDR1 with DIV, a coil‐coiled domain from *Bacillus subtilis* DivIVA that forms constitutive dimer/oligomers [41]. Replacement of the DDR1 ligand binding domain with DIV promotes spontaneous DDR1 autophosphorylation and activation [41]. As a control, we used a construct that has mutations in the DIV coil‐coiled domain (*mDIV‐DDR1*) which disrupts DIV self‐assembly ability [41]. We confirmed the expression and activation status of DDR1 in transfected cells by immunoblotting. As expected, cells transfected with the constitutively active *DDR1* gene showed high levels of DDR1 phosphorylation at Y792 (Figure S7). In contrast, and in keeping with prior reports, cells transfected with the mutant *DDR1* showed lower but detectable levels of DDR1 phosphorylation (Figure S7). We then assessed the impact of DDR1 activation on CIN in DG75 cells. To do this, we counted chromosomes in metaphase spreads of cell populations transiently transfected either with the *DIV* or *mDIV* construct. DG75 cells expressing constitutively activated *DDR1* had significantly higher numbers of hyperdiploid cells compared to controls (Figures 7 and S8). We also observed increased numbers of hyperdiploid cells following *mDIV* transfection, but to a lesser degree than in DIV‐expressing cells, consistent with reduced DDR1 activation in *mDIV*‐expressing cells (Figure 7). We conclude that an activated DDR1 receptor can induce CIN in B cells.

## 4 Discussion

We have shown that DDR1 is overexpressed in a subset of DLBCL, including both GC and non‐GC subtypes. Meta‐analysis revealed that *DDR1* expression was tightly linked to *collagen VI* expression, which we showed was intimately associated with DDR1‐expressing tumour cells in tumour biopsies. In keeping with previous reports [23, 42], the meta‐analysis also revealed that genes positively correlated with *DDR1* were enriched genes involved in proliferation and protection from apoptosis. However, genes negatively correlated with *DDR1* were enriched for genes associated with mitotic spindle functions, including chromosome segregation. Strikingly, three separate aneuploidy signatures were enriched among genes correlated with *DDR1*. Moreover, we were able to induce aneuploidy experimentally in a *TP53*‐mutant B‐cell lymphoma cell line following the overexpression of DDR1.

The kinesin motor protein, CENPE, was downregulated following DDR1 activation in both untransformed GC B cells and B‐cell lymphoma lines. During mitosis, CENPE localises to kinetochores, linking chromosomes to microtubules of the mitotic spindle [43, 44]. Reduced CENPE expression causes chronic misalignment of one or a few chromosomes at the spindle poles [43, 44]. CENPE also has critical functions in the SAC providing a fail‐safe mechanism to prevent chromosome mis‐segregation and the development of aneuploidy [45]. The SAC is weakened in cells which have reduced CENPE expression, and *CENPE*+/− cells can enter anaphase in the presence of one or a few misaligned chromosomes, resulting in aneuploid progeny [43, 44, 45]. While we observed an inverse correlation between *DDR1* and *Ki67* expression in wild‐type tumours, this was not observed in *TP53*‐mutant tumours. Thus, the downregulation of CENPE is unlikely to be due to an indirect effect of DDR1 on proliferation in *TP53*‐mutant tumours. This is further supported by our data showing that ectopic expression of DDR1 in GC B cells induces transcriptional changes consistent with enhanced proliferation.

Although we have not directly studied the mechanism by which DDR1 regulates CENPE expression, it is plausible that DDR1 could influence CENPE indirectly through the PI3K/AKT signalling pathway. DDR1 activation is known to modulate this pathway [46], which can stabilise and enhance the activity of transcription factors such as FoxM1 and MYC [47, 48, 49]. These factors, which are often aberrantly expressed in DLBCL [50, 51], are known regulators of *CENPE* transcription [52, 53], and may serve as intermediaries in DDR1's effect on CENPE downregulation.

In certain contexts, increased whole‐chromosome mis‐segregation resulting from reduced CENPE expression can suppress tumour formation [54]. Cells in which tumour suppression occurs following reduced CENPE expression were shown to have already pre‐existing, elevated basal levels of chromosome mis‐segregation that were further exacerbated by reduced CENPE expression. In contrast, tumours that arose as a consequence of transforming events that do not induce chromosomal instability were unaffected by CENPE‐dependent chromosome mis‐segregation [54]. These findings suggest that low rates of chromosome mis‐segregation can promote tumourigenesis, whereas mis‐segregation of more than a few chromosomes leads to cell death and tumour suppression [54].

Our analysis of publicly available data shows that among cases with high levels of *DDR1*, a higher frequency of SCNA was only observed in *TP53*‐mutant, but not in *TP53* wild‐type, tumours, suggesting that *TP53* inactivation might be required to inhibit cell cycle arrest and allow the propagation of chromosome imbalances to daughter cells. During a normal cell cycle, aneuploidy is prevented by the function of the mitotic spindle checkpoint, a p53‐dependent G1 checkpoint and an additional G2 checkpoint [40].

DLBCL is reported to be heterogeneously aneuploid, and CIN is associated with tumour aggressiveness and disease evolution in non‐Hodgkin lymphomas [5, 55, 56, 57]. For example, more aggressive DLBCLs were shown to harbour centrosome aberrations in 41.8% of cases compared to 25.5% of more indolent lymphomas [5]. Moreover, higher‐frequency chromosome segregation defects have been shown to be associated with a decrease in overall survival, and an increase in tumour invasiveness and relapse after treatment in DLBCL patients [6]. A more recent publication showed that ≥ 3 chromosomal abnormalities were significantly associated with inferior overall survival in DLBCL [7].

The overexpression of DDR1 in DLBCL might be a consequence of the altered activity of cell signalling pathways, including NF‐κB and AP‐1, which are known to regulate DDR1 expression and which are also aberrantly activated in DLBCL [18].

Lymphoma development has been causally linked to chronic inflammation [58]. DLBCL associated with chronic inflammation is a distinct entity in the most recent lymphoma classification [59]. Our finding that CENPE is downregulated by DDR1 provides one potential mechanistic explanation for this association. Our observations also raise the possibility that therapeutic agents that further increase CIN might be a promising therapeutic approach in DLBCL [54].

## Author Contributions

**Sandra Margielewska‐Davies:** conceptualization (equal), investigation (lead), validation (equal), visualization (equal), writing – original draft (equal), writing – review and editing (equal). **Matthew Pugh:** conceptualization (equal), data curation (equal), investigation (equal), methodology (equal). **Eszter Nagy:** conceptualization (equal), investigation (equal), methodology (equal), supervision (equal), validation (equal). **Ciara I. Leahy:** investigation (equal), methodology (equal), visualization (equal). **Maha Ibrahim:** formal analysis (equal), investigation (equal), methodology (equal). **Eanna Fennell:** formal analysis (equal), investigation (equal), validation (equal), visualization (equal). **Aisling Ross:** investigation (equal). **Jan Bouchal:** resources (equal). **Lauren Lupino:** conceptualization (equal), formal analysis (equal), investigation (equal), methodology (equal), validation (equal). **Matthew Care:** resources (equal), software (equal). **Reuben Tooze:** resources (equal). **Gary Reynolds:** formal analysis (equal), investigation (equal), visualization (equal). **Zbigniew Rudzki:** formal analysis (equal). **Wenbin Wei:** data curation (equal), formal analysis (equal), validation (equal). **William Simmons:** resources (equal). **Vikki Rand:** resources (equal). **Kelly Hunter:** investigation (equal). **John J. Reynolds:** investigation (equal). **Grant S. Stewart:** supervision (equal). **Katerina Bouchalova:** resources (equal). **Iona J. Douglas:** methodology (equal). **Katerina Vrzalikova:** conceptualization (equal), formal analysis (equal), investigation (equal), methodology (equal), supervision (equal), validation (equal), visualization (equal), writing – review and editing (equal). **Paul G. Murray:** conceptualization (lead), formal analysis (equal), funding acquisition (lead), investigation (supporting), project administration (lead), supervision (lead), validation (equal), visualization (equal), writing – original draft (equal), writing – review and editing (equal).

## Conflicts of Interest

The authors declare no conflict of interest.

## Supporting information


Figures S1–S8.

Tables S1–S5.

Data S1.

## Data Availability

The datasets generated and/or analysed in this study are available in Supporting Information files or from the corresponding author on reasonable request.
