# Supplementary material for: The Overexpression of Collagen Receptor DDR1 is Associated With Chromosome Instability and Aneuploidy in Diffuse Large B‐Cell Lymphoma
Source: J Cell Mol Med. 2025 May 22;29(10):e70318. doi: 10.1111/jcmm.70318 (PMC12096173; doi:10.1111/jcmm.70318)
Supplement: Supplementary file 1 — Figures S1–S8. [file JCMM-29-e70318-s003.docx]

**Supplementary Figure 1**

**A**

**DG75**


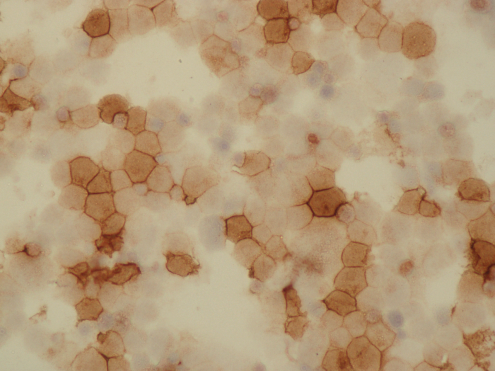

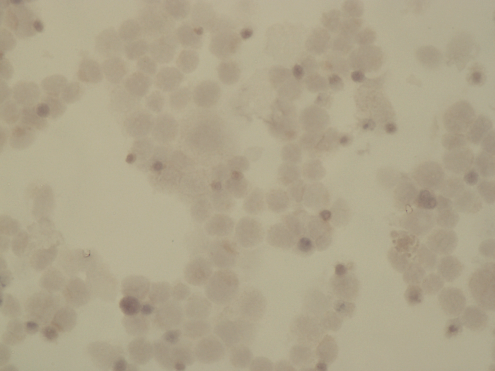


**Empty vector**

**DDR1α-transfected**

**B**

**Empty vector**

**DDR1α**

**DDR1**

**β-actin**


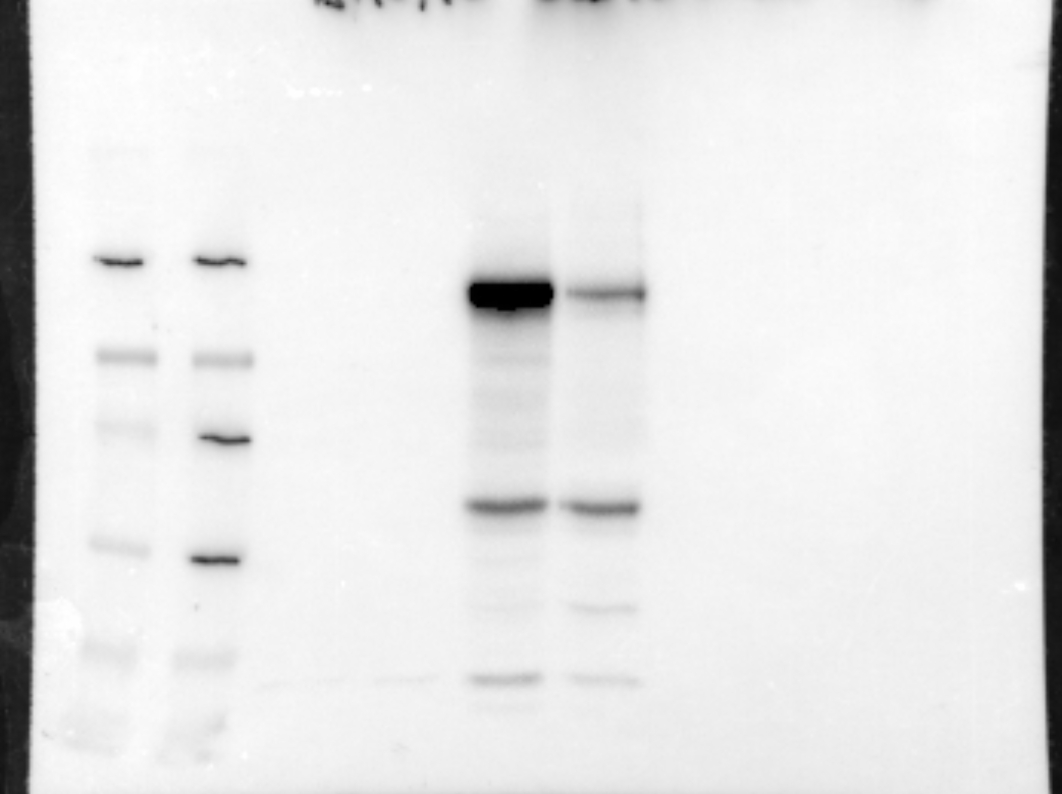

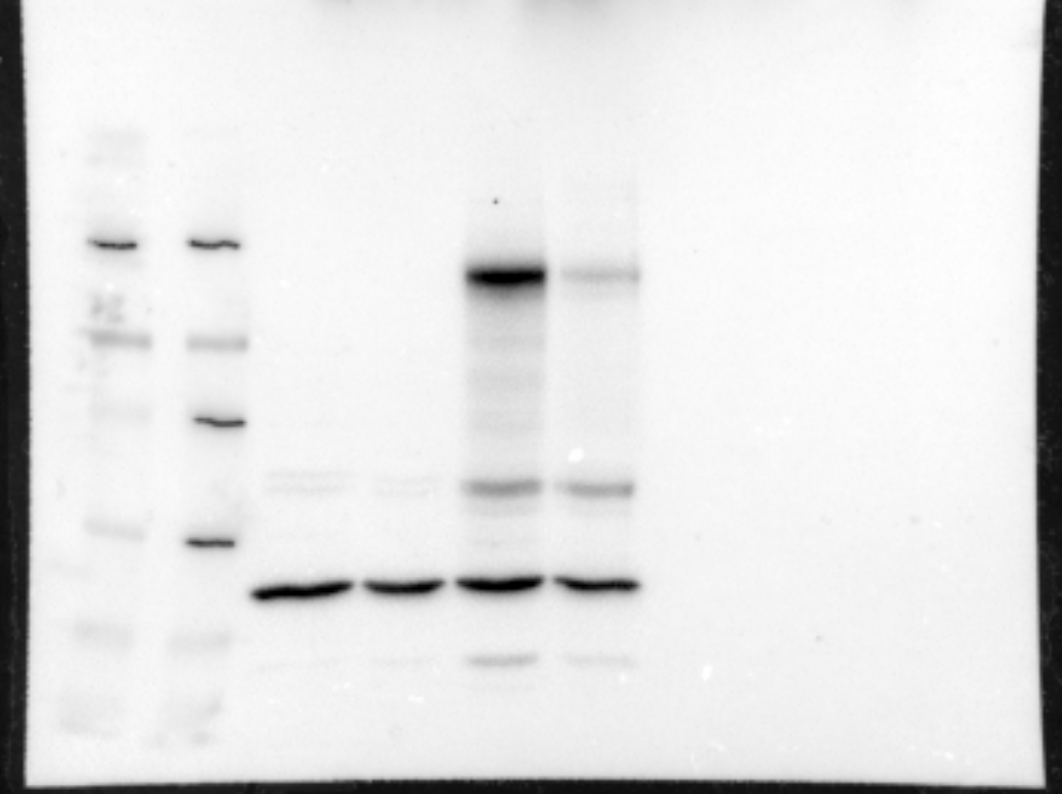


**125kDa**

**45kDa**

**DG75**

**Supplementary Figure 1**


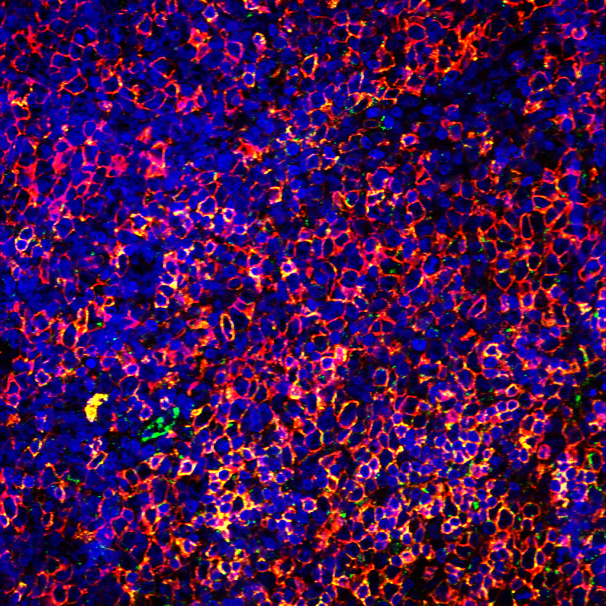

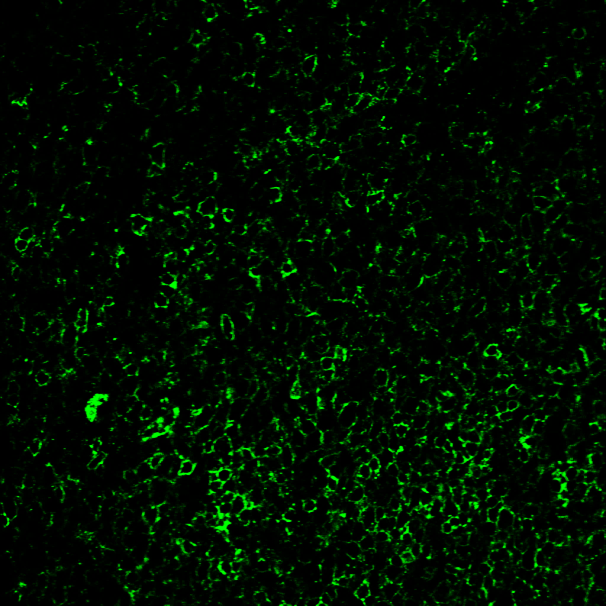

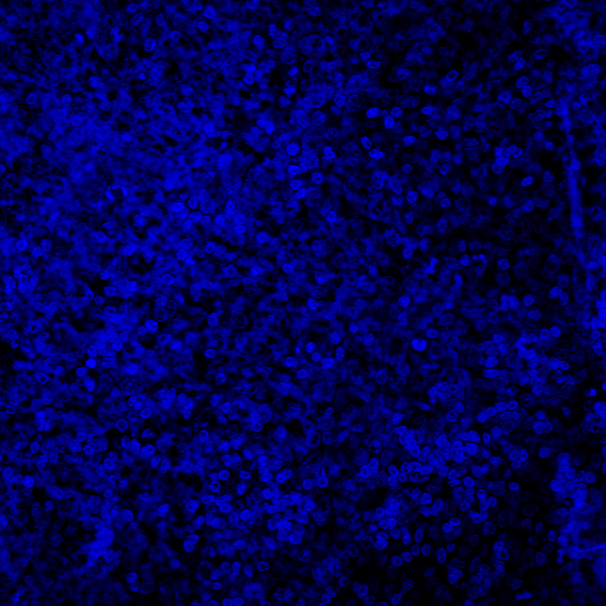

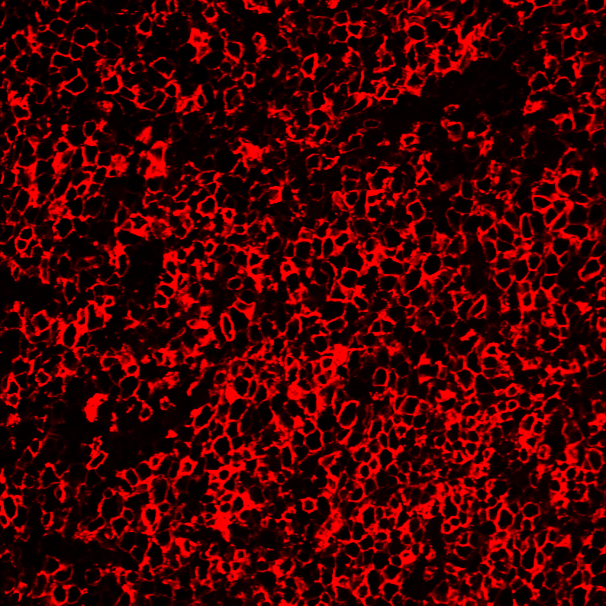


**C**

**DLBCL**

**DAPI**

**CD20**

**merged**

**DDR1**

**Supplementary Figure 1**


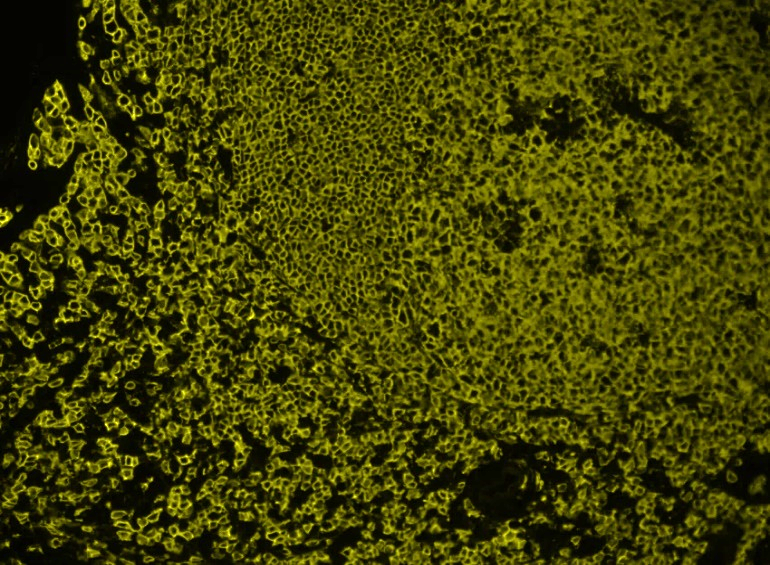

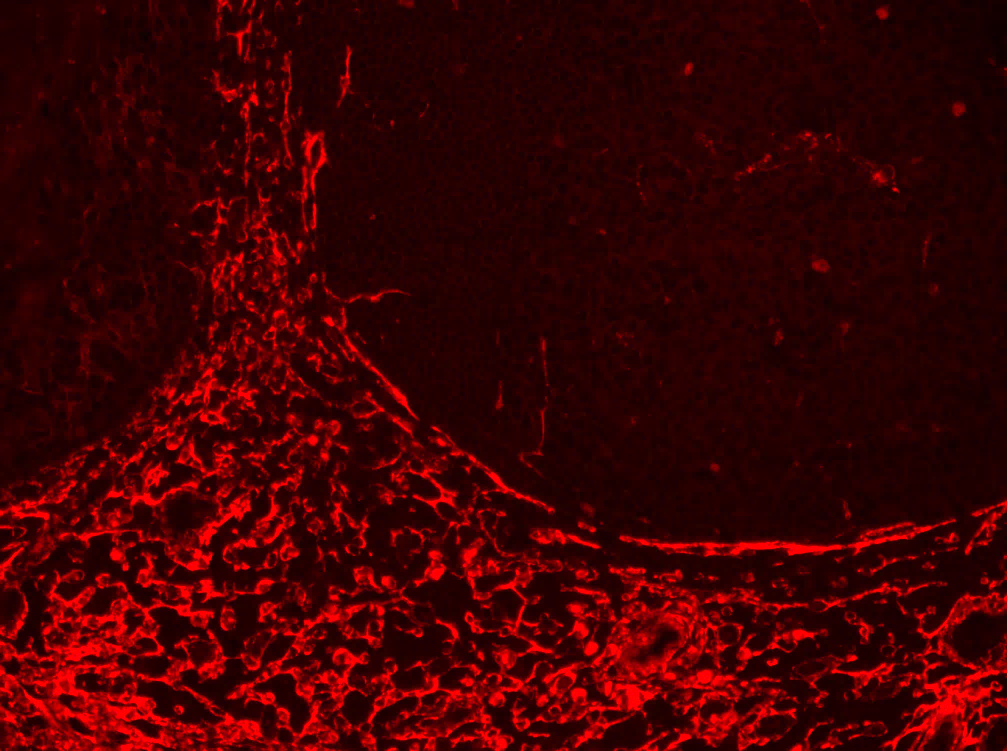

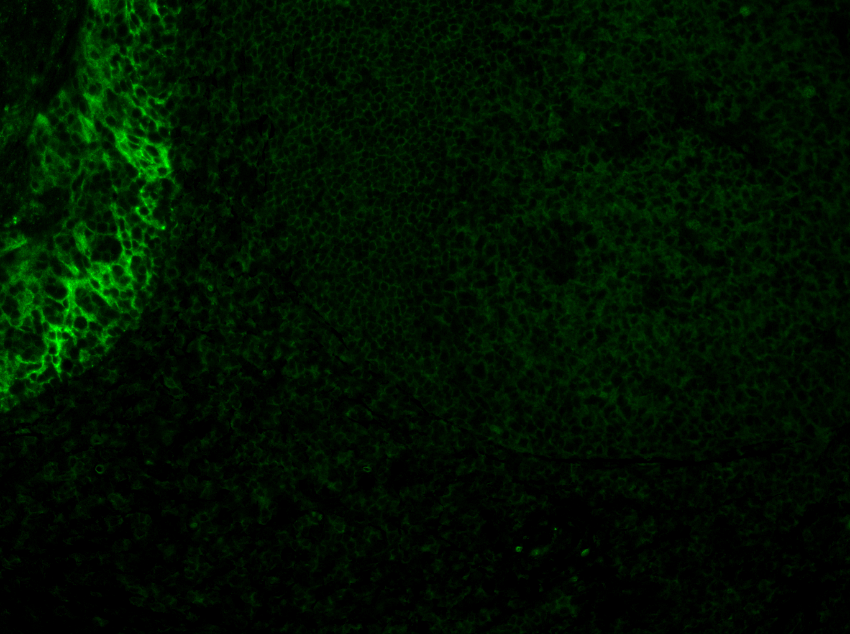

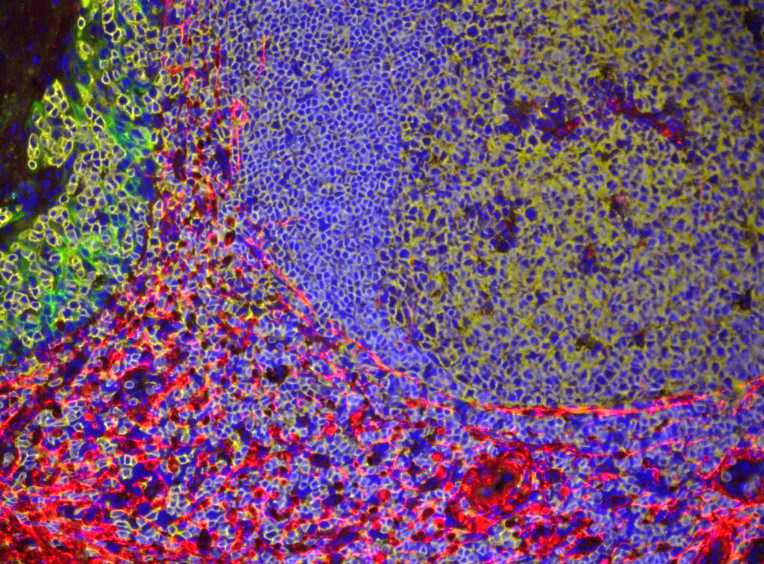


**Tonsil**

**D**

EP

EP

EP

EP

GC

GC

GC

GC

**COLVI**

**DDR1**

**CD20**

**merged**

**Supplementary Figure 1**


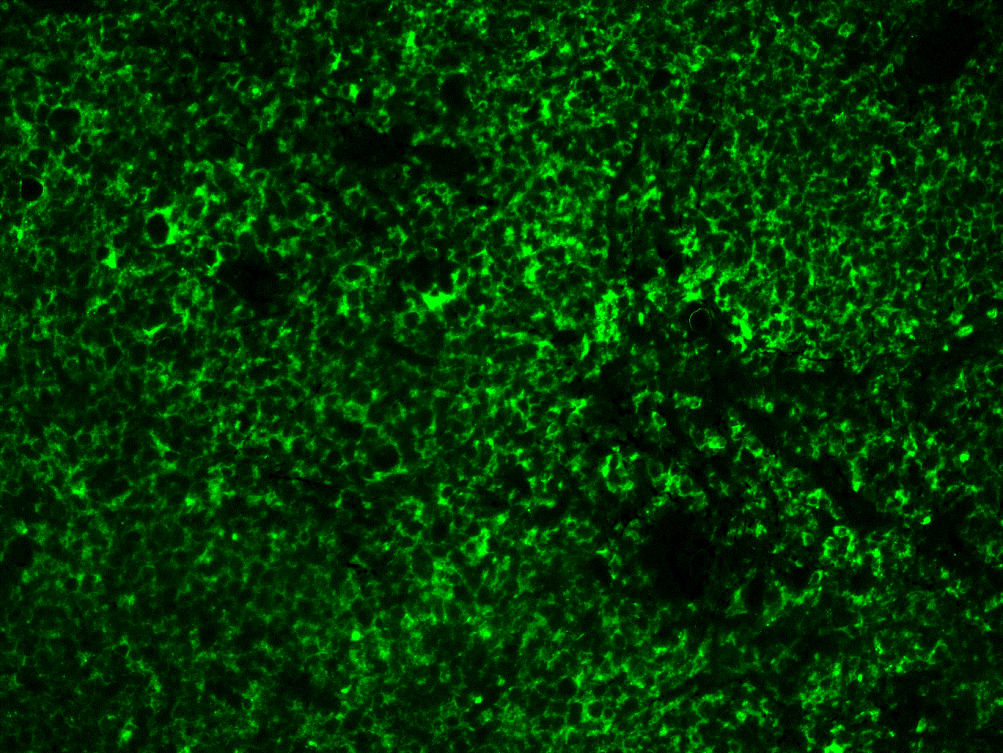

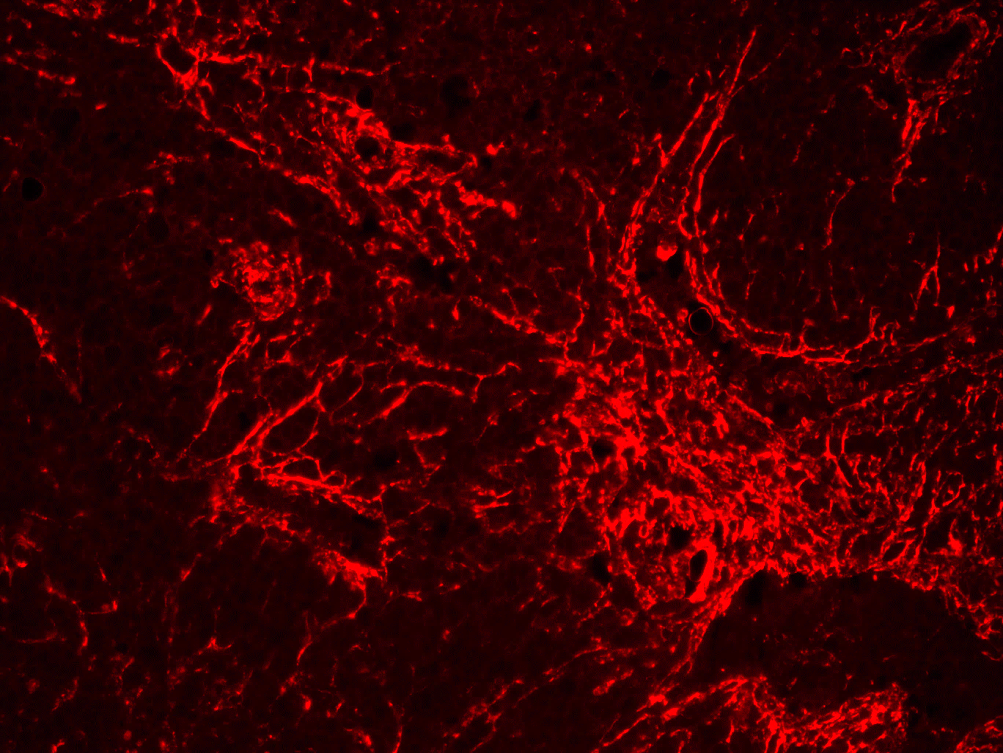

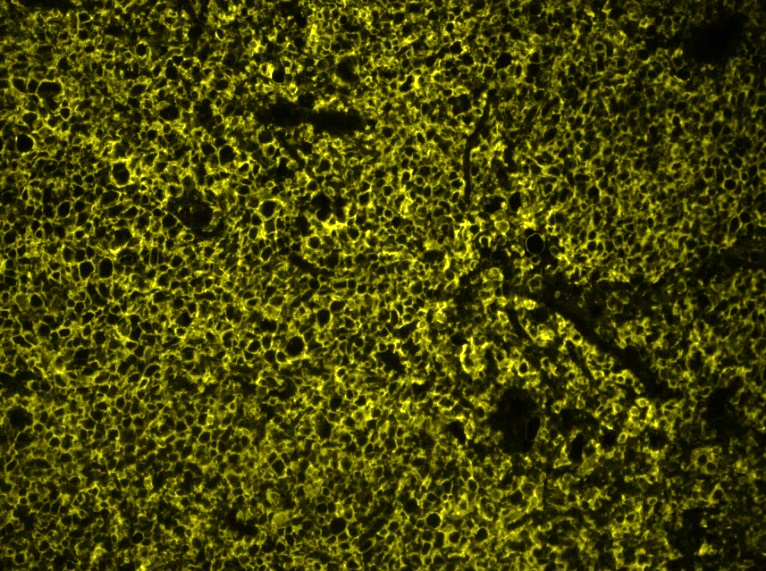

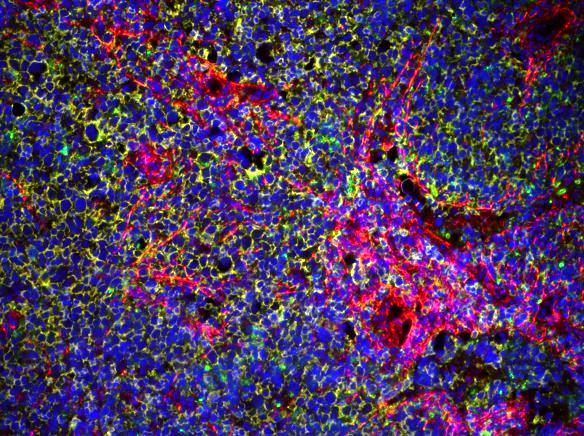


**E**

**DLBCL**

**DDR1**

**COLVI**

**merged**

**CD20**

**Supplementary Figure 1**

**F**

p=0.41

**merged**


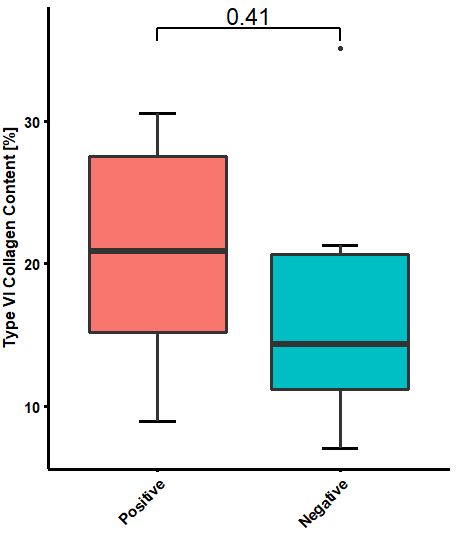


**Supplementary Figure 2**

**A**

**
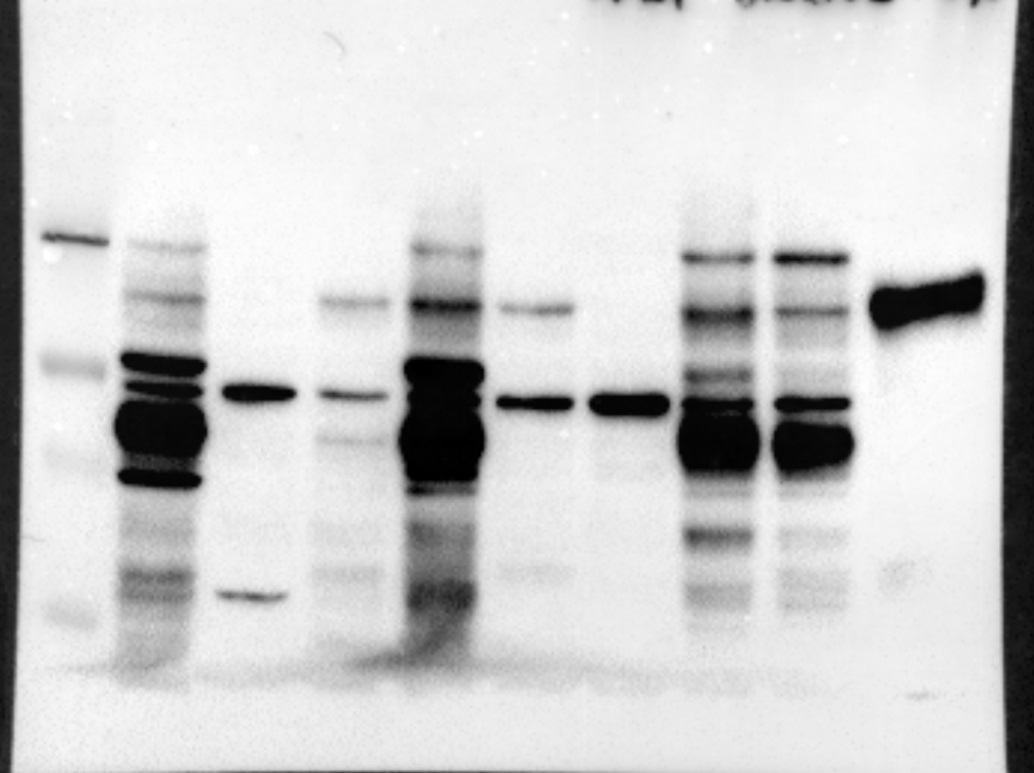
**

DDR1

125kDa

**
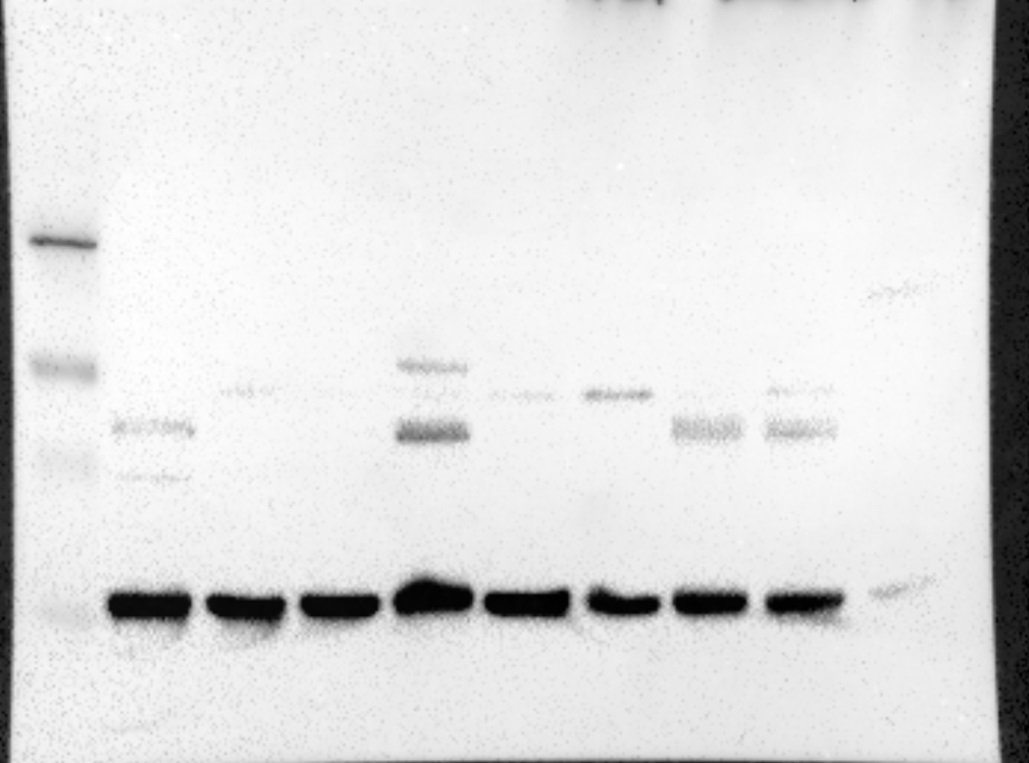
**

55kDa

β-tubulin

Farage

HT

Karpas

OCI-LY1

OCI-LY7

U2932

OCI-LY3

BJAB

**B**

**Supplementary Figure 3**

**
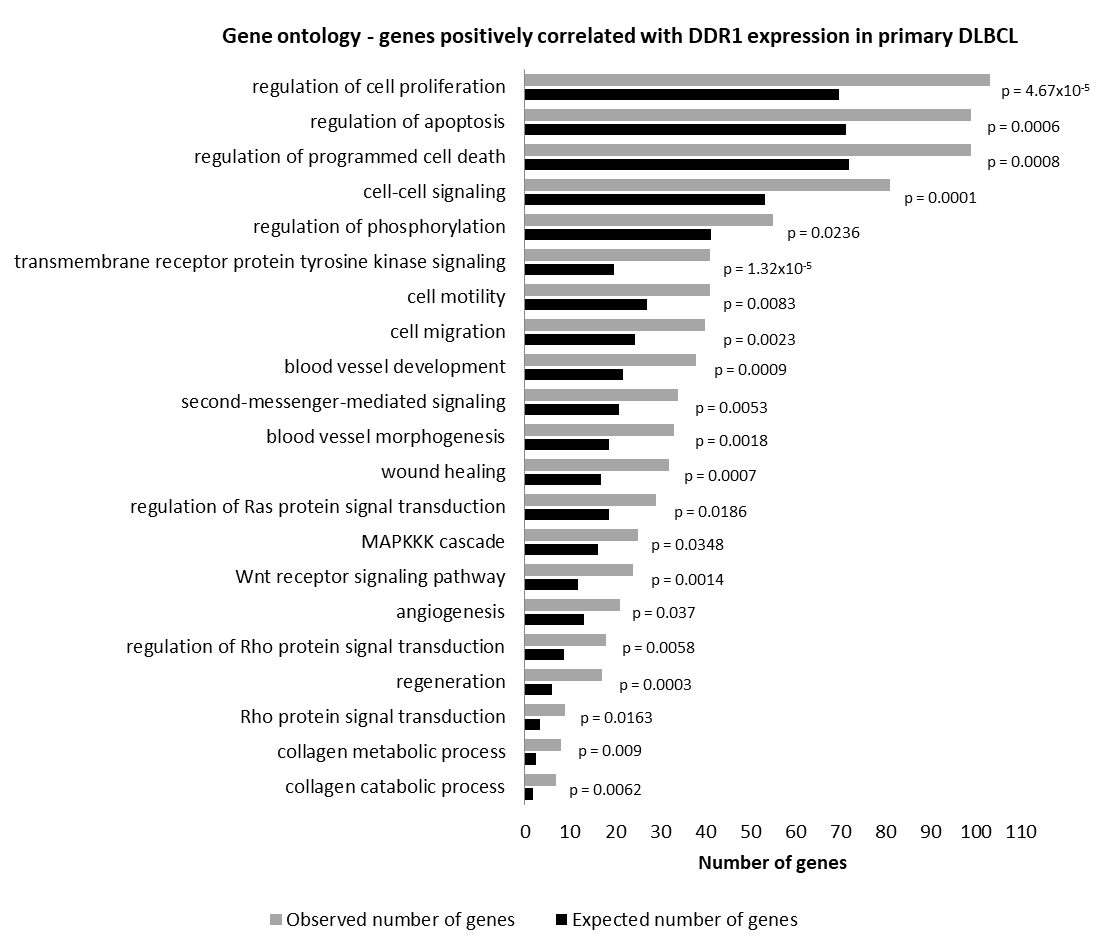
**

**A**

**B**

**
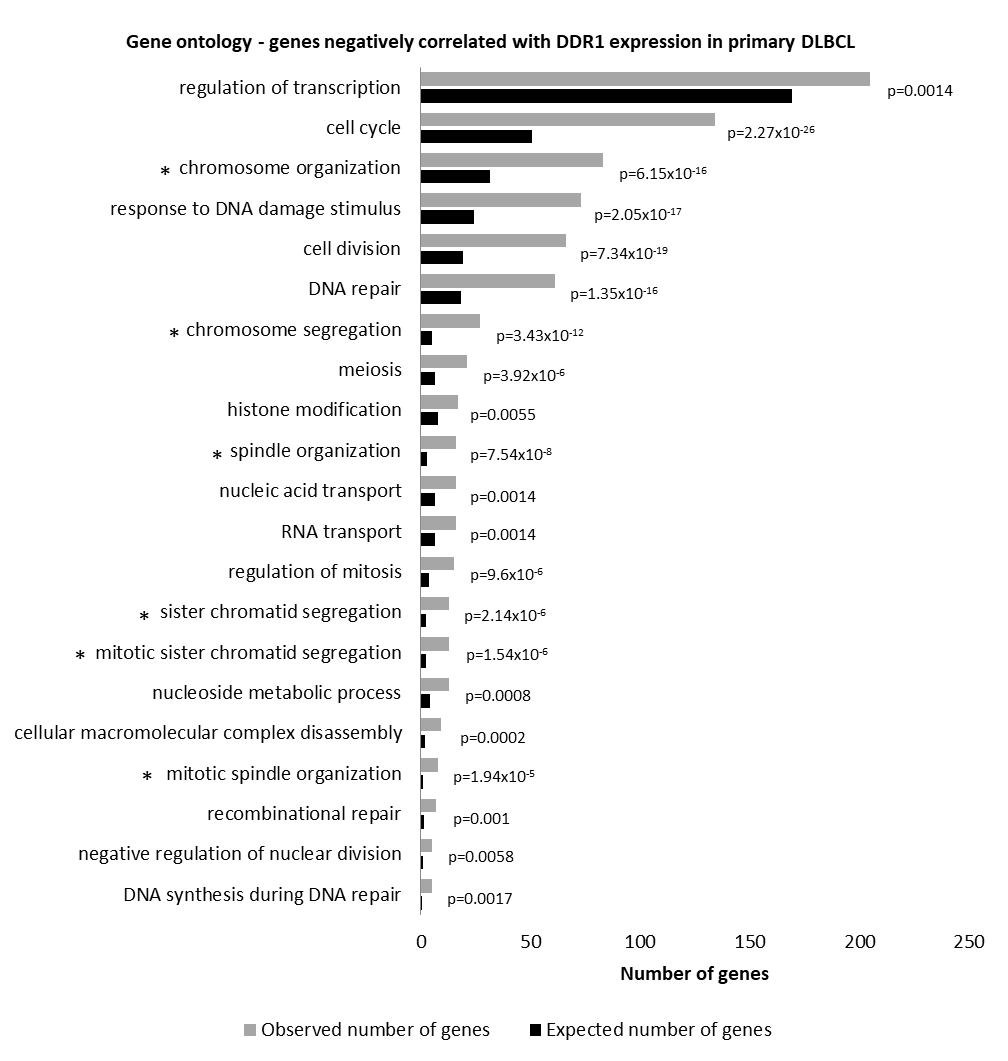
**

**Supplementary Figure 4**

**
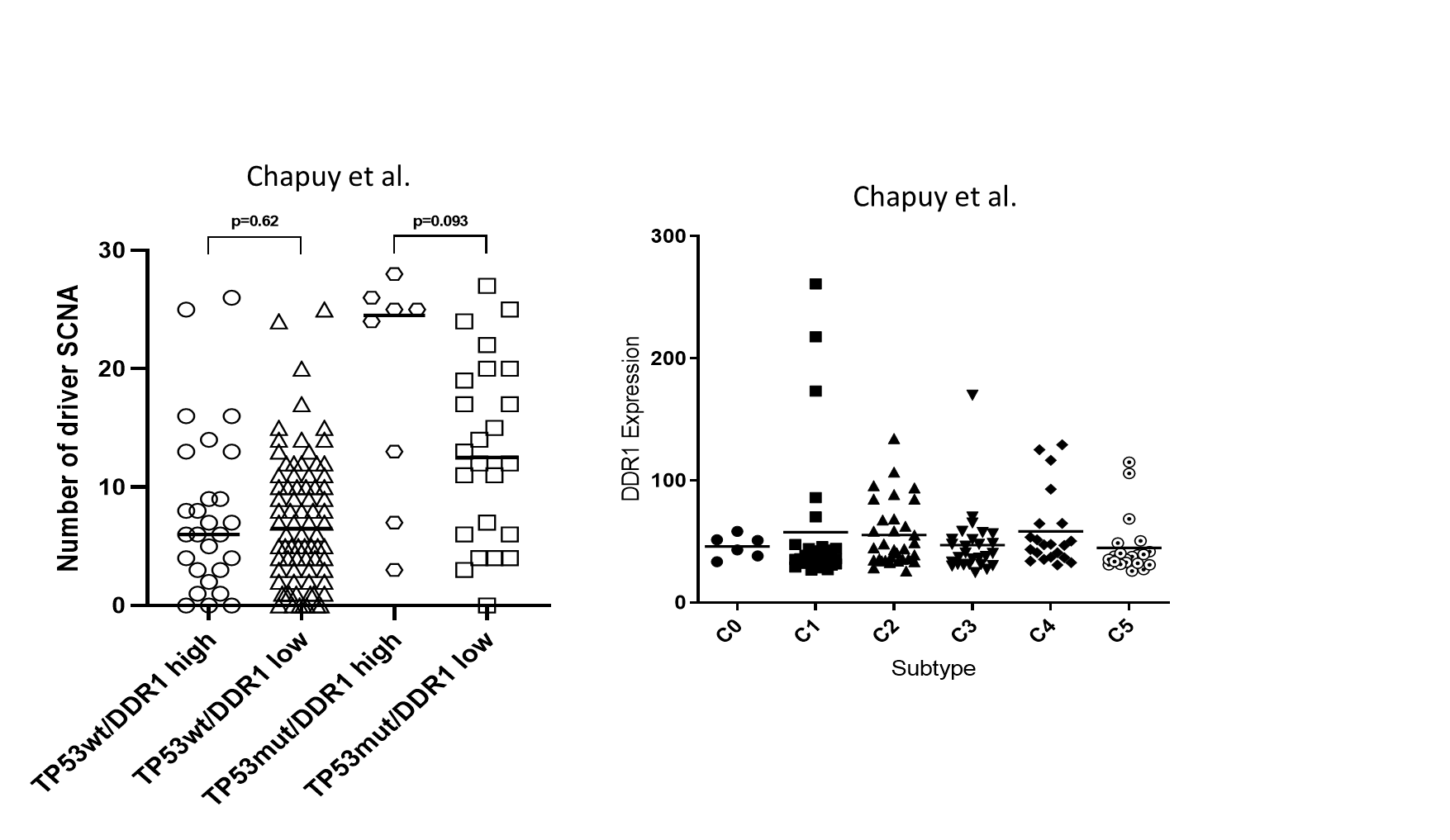
**

**Supplementary Figure 5**

**A**


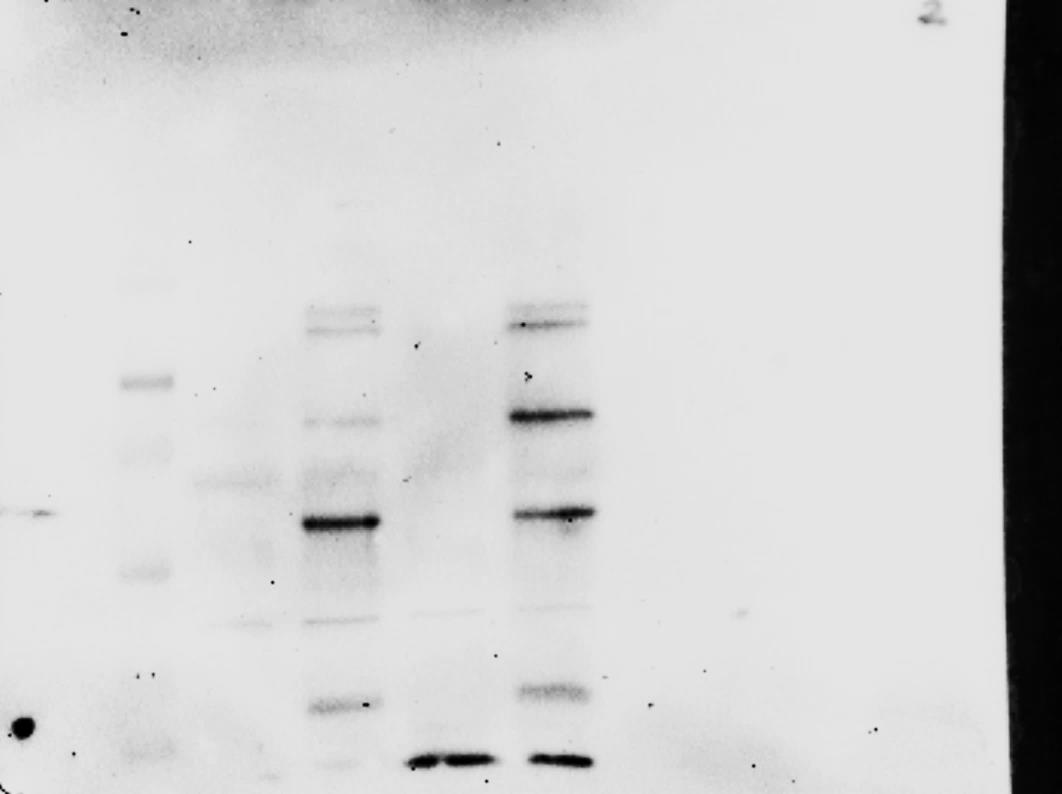

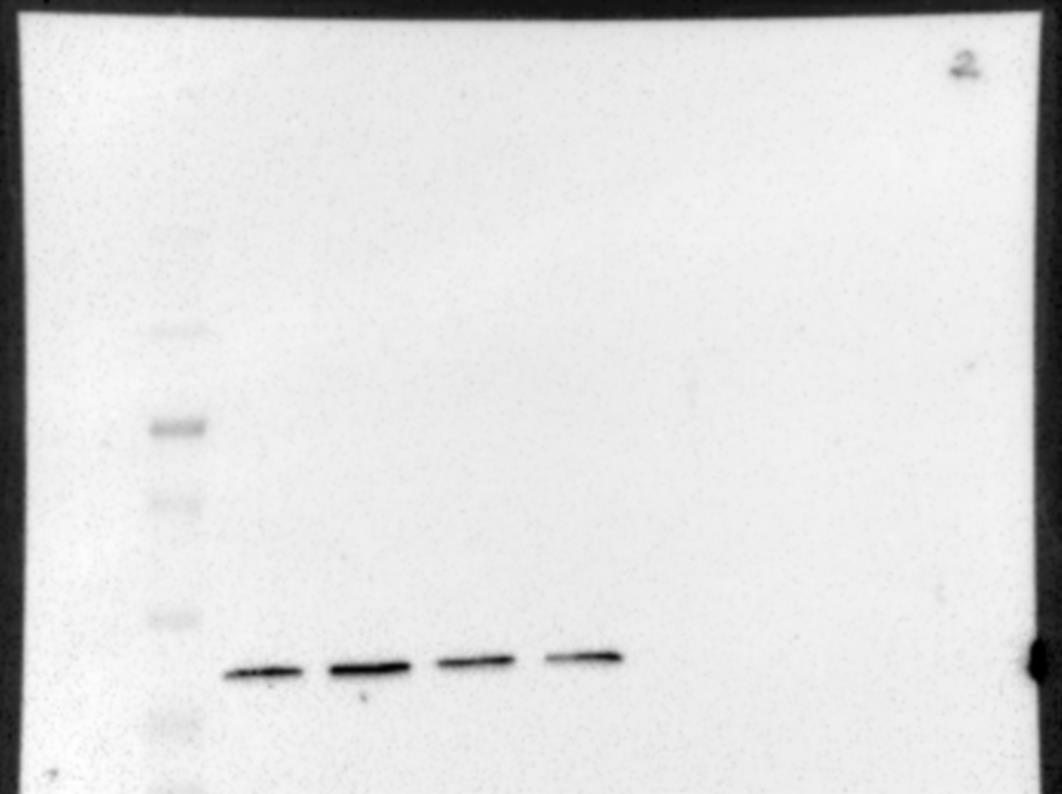


**T24-EV**

**T24-DDR1**

**DDR1**

**β-actin**

**125kDa**

**45kDa**

**B**

**Supplementary Figure 5**

**
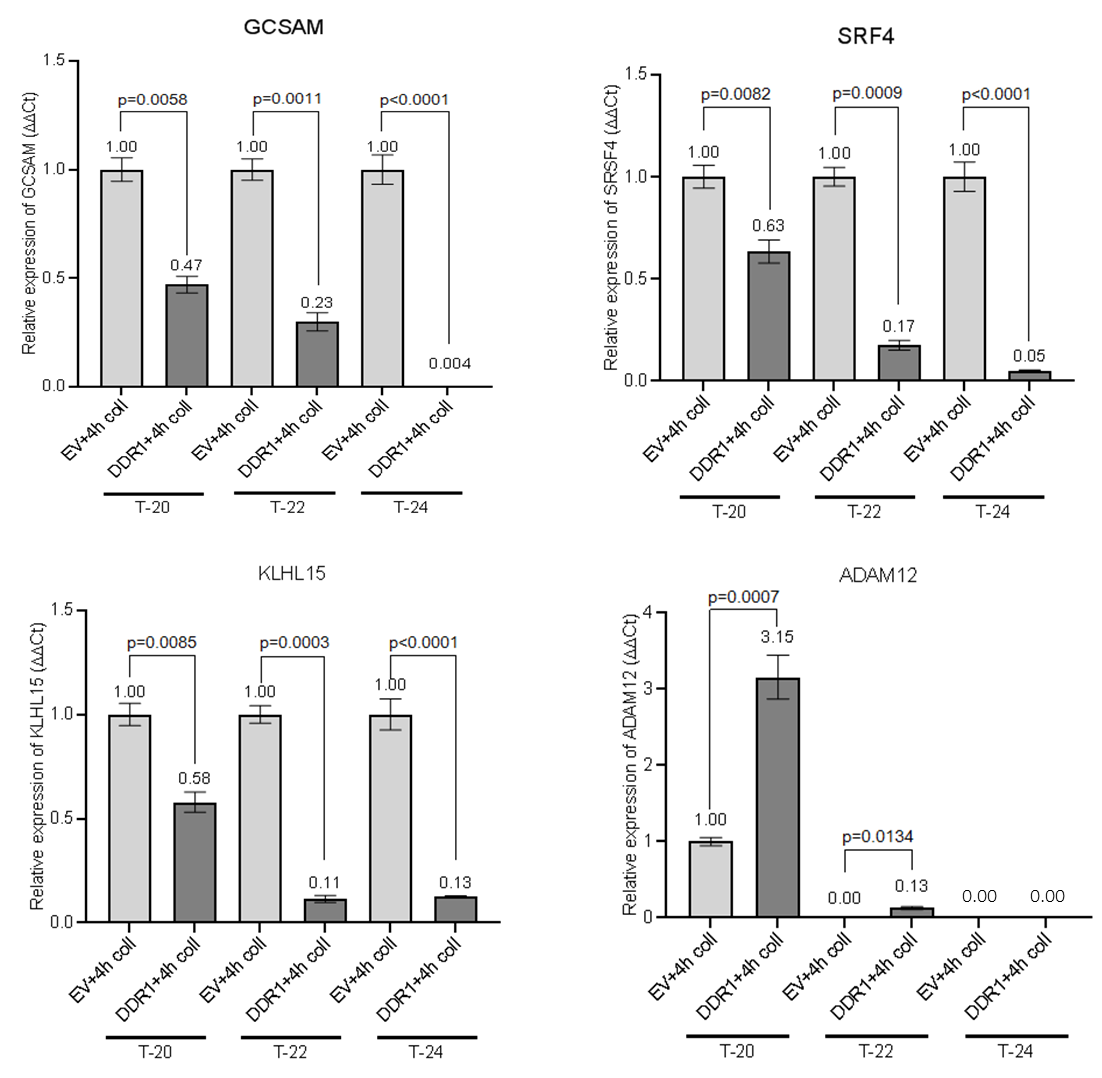
**

**SRSF4**

**Supplementary Figure 5**

**C**


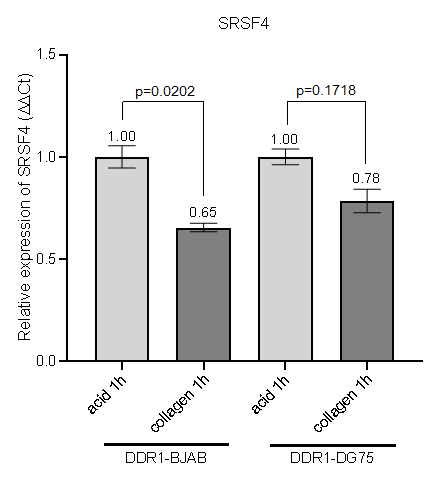

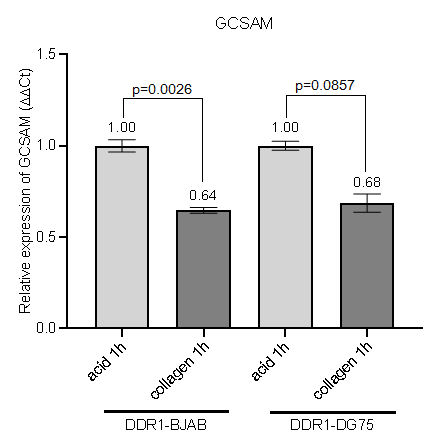


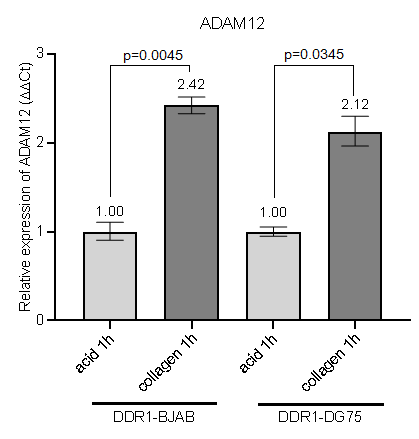

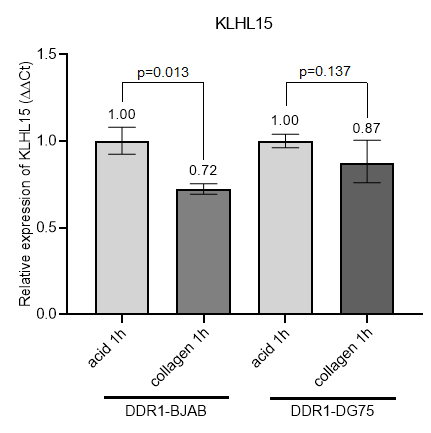


**
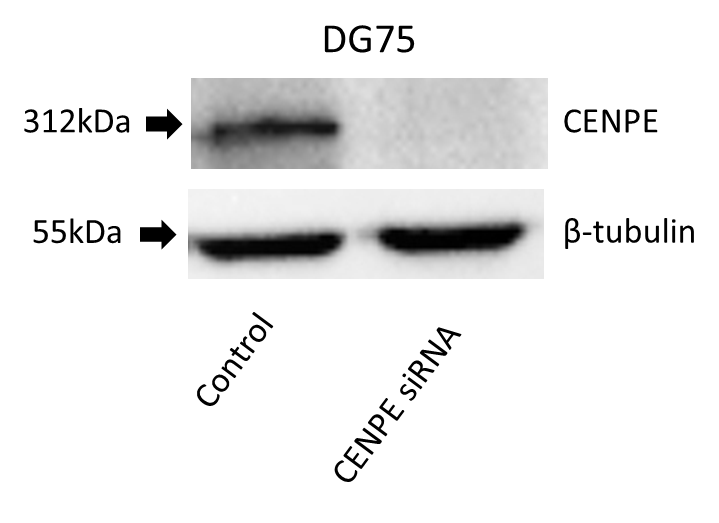
**

**Supplementary Figure 6**

**A**

**
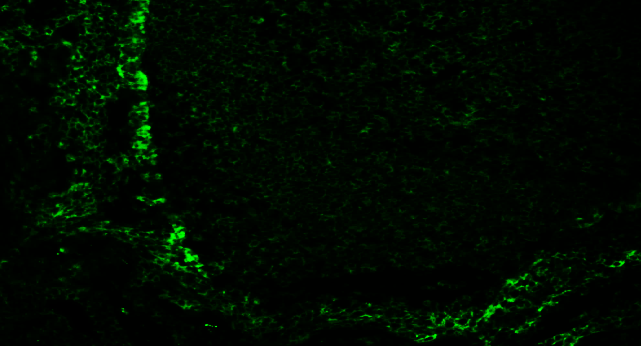

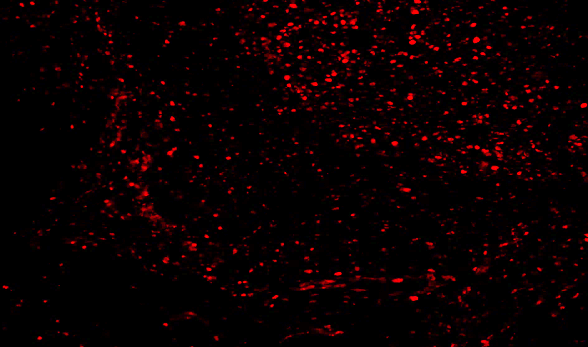

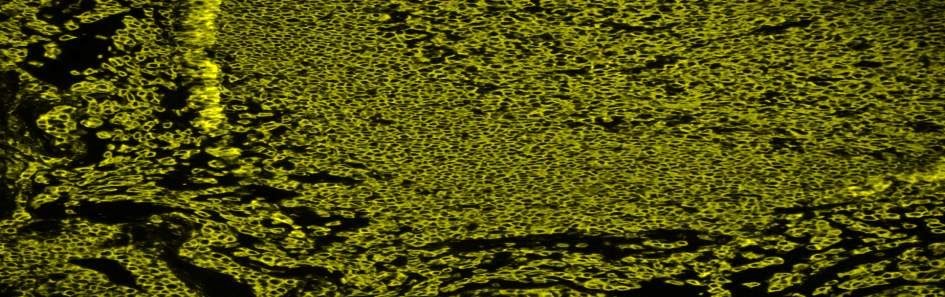

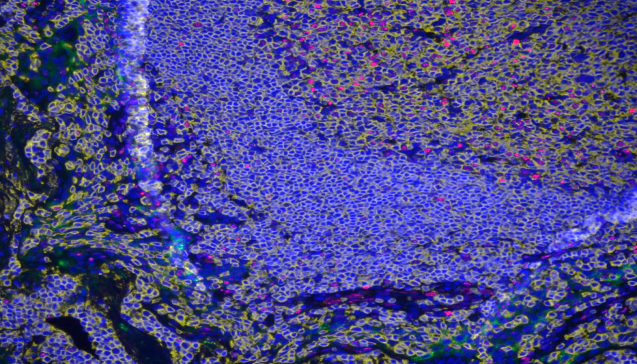

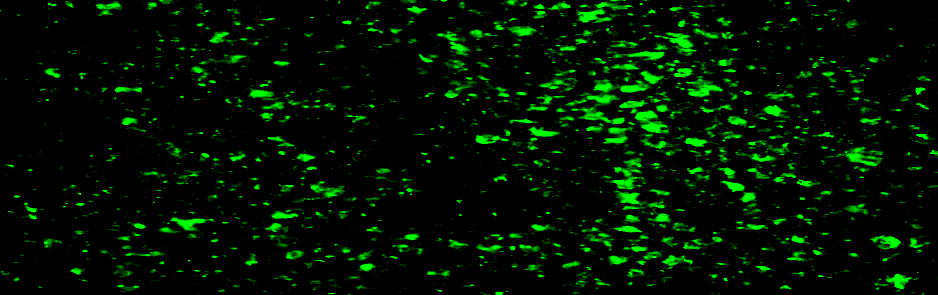

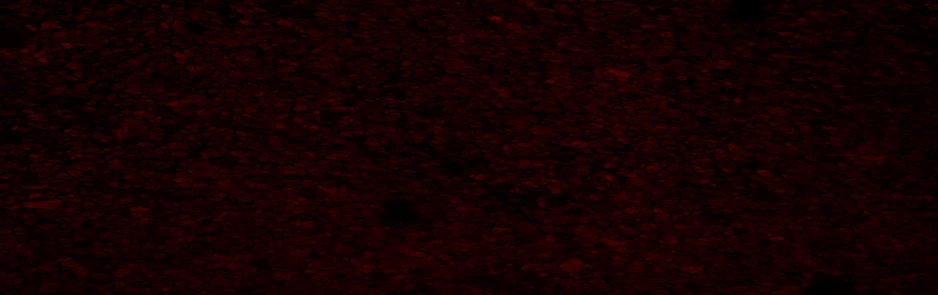

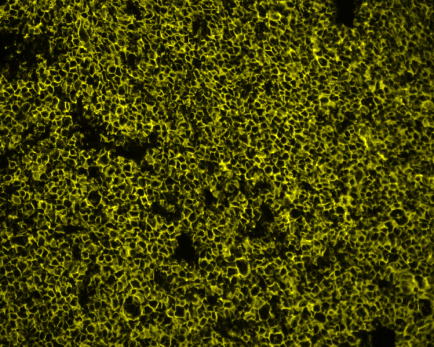

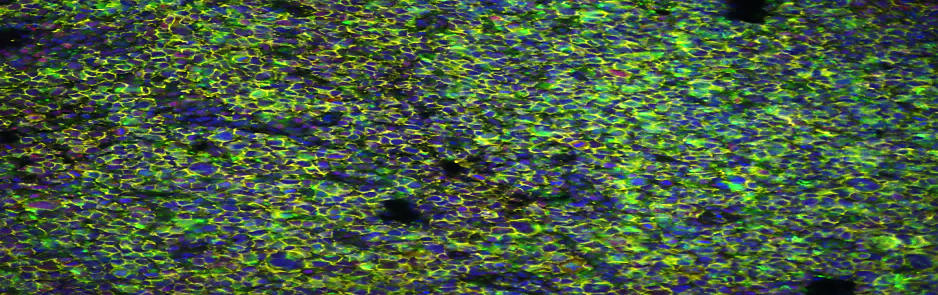

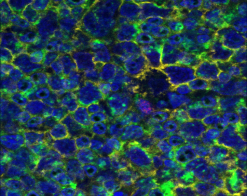

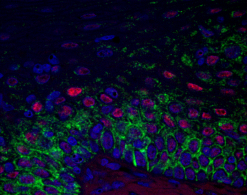
**

**Supplementary Figure 6**

**GC**

**B**

**Tonsil**

**DDR1**

**CD20**

**Merged**

**CENPE**

**DLBCL**

**DDR1**

**CD20**

**Merged**

**CENPE**

**Supplementary Figure 7**

**
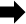

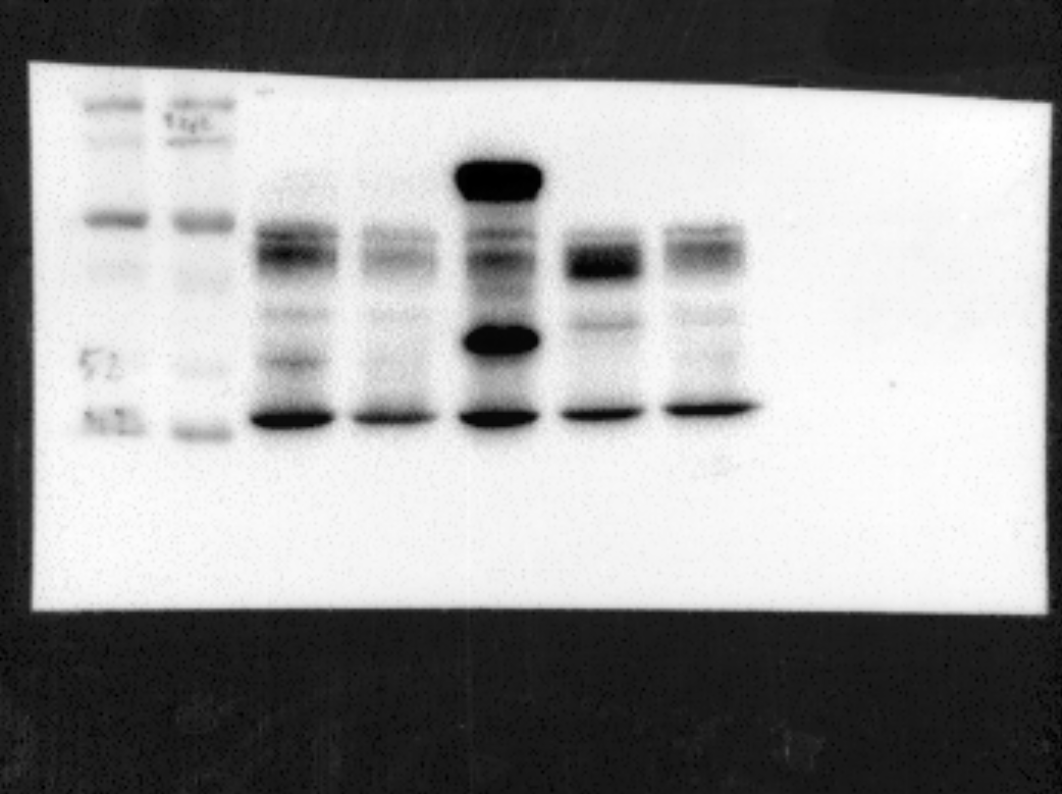

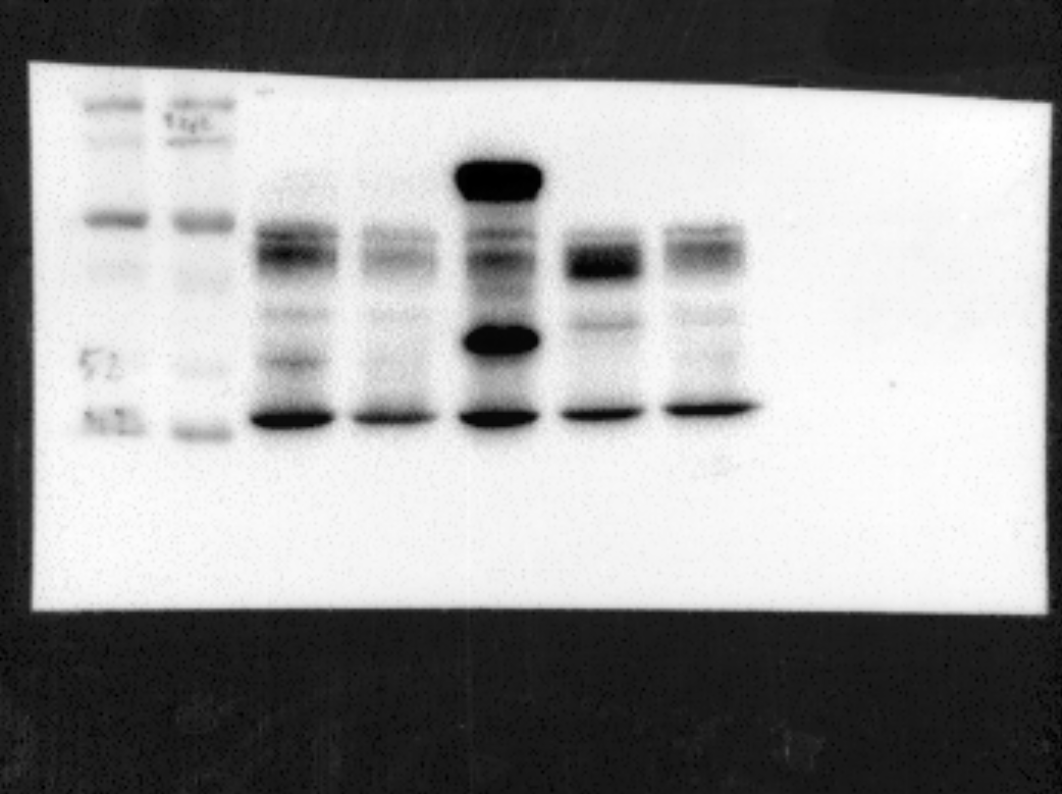

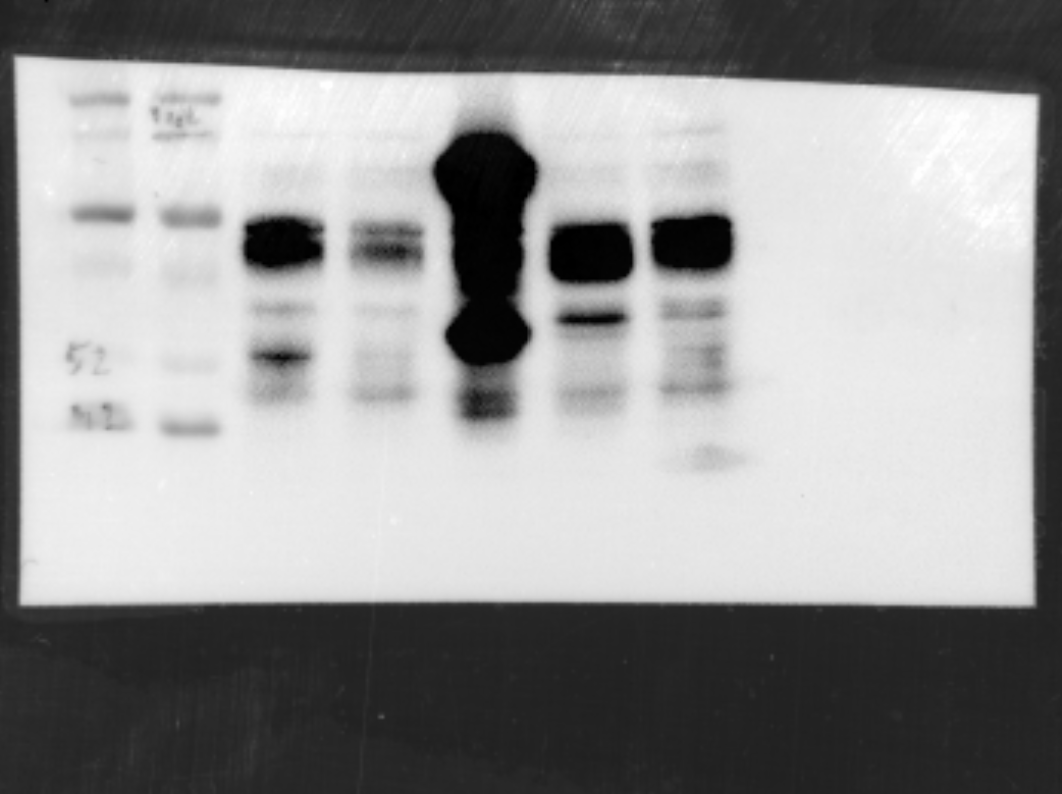

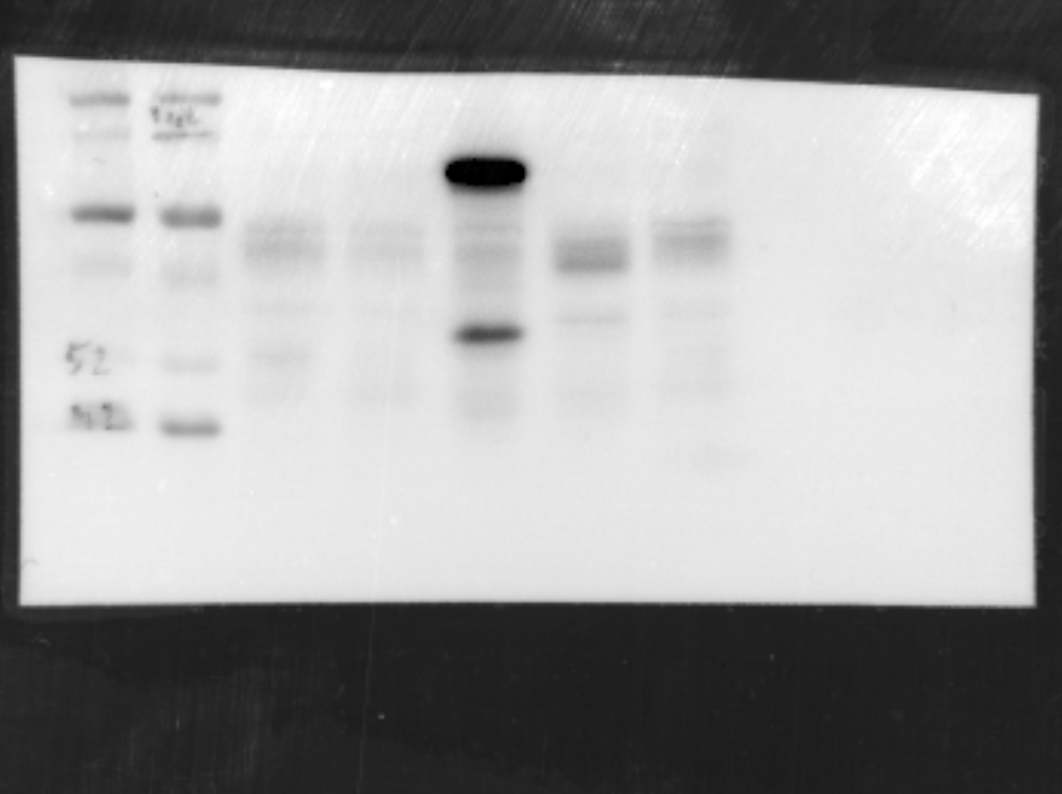

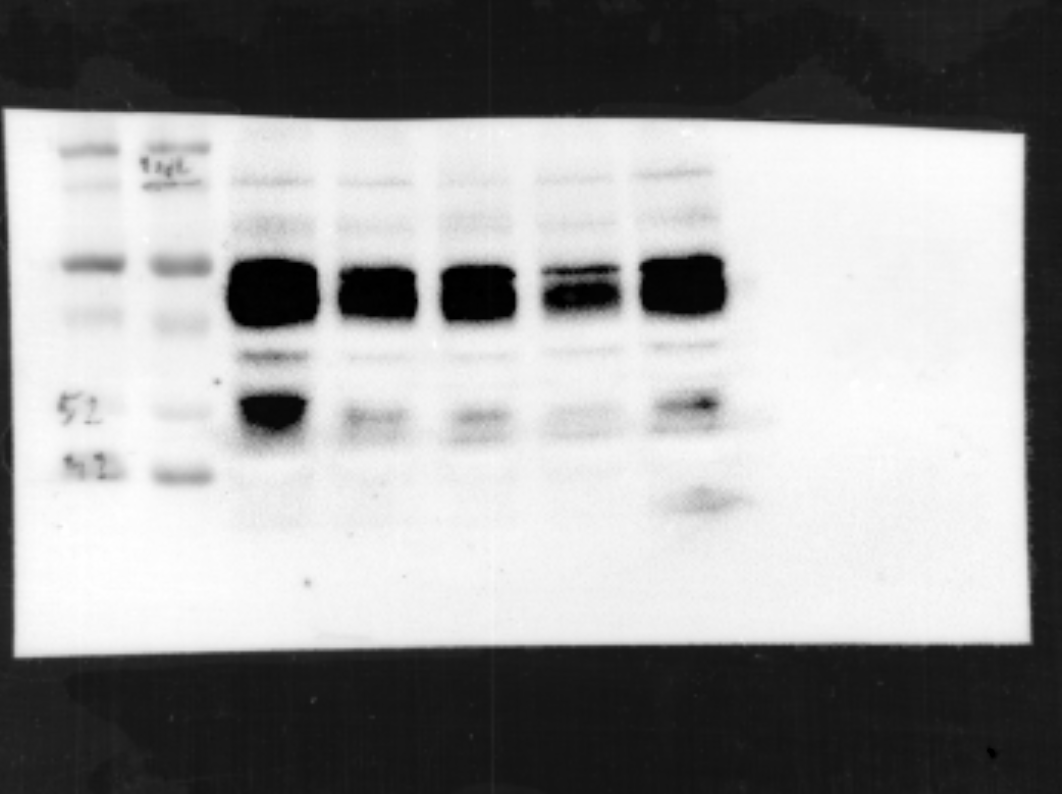

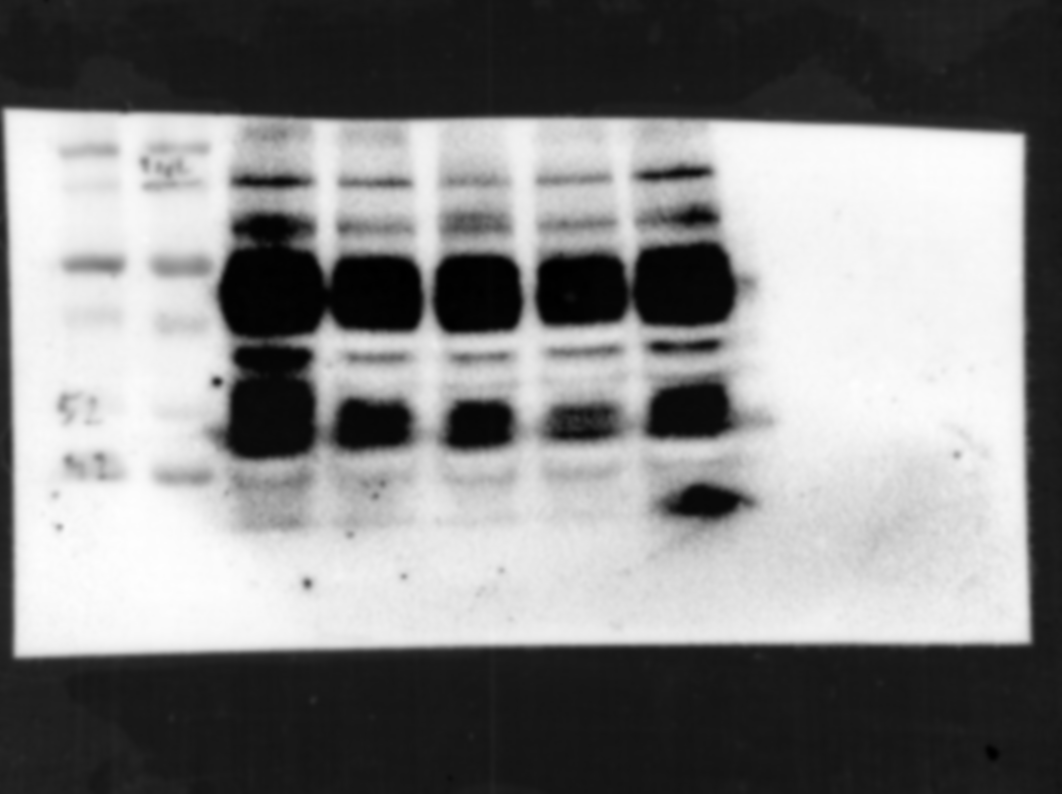
**

DG75

DIV

DDR1α

mDIV

125kDa

pDDR1

125kDa

tDDR1

45kDa

β-actin

**Supplementary Figure 8**

**
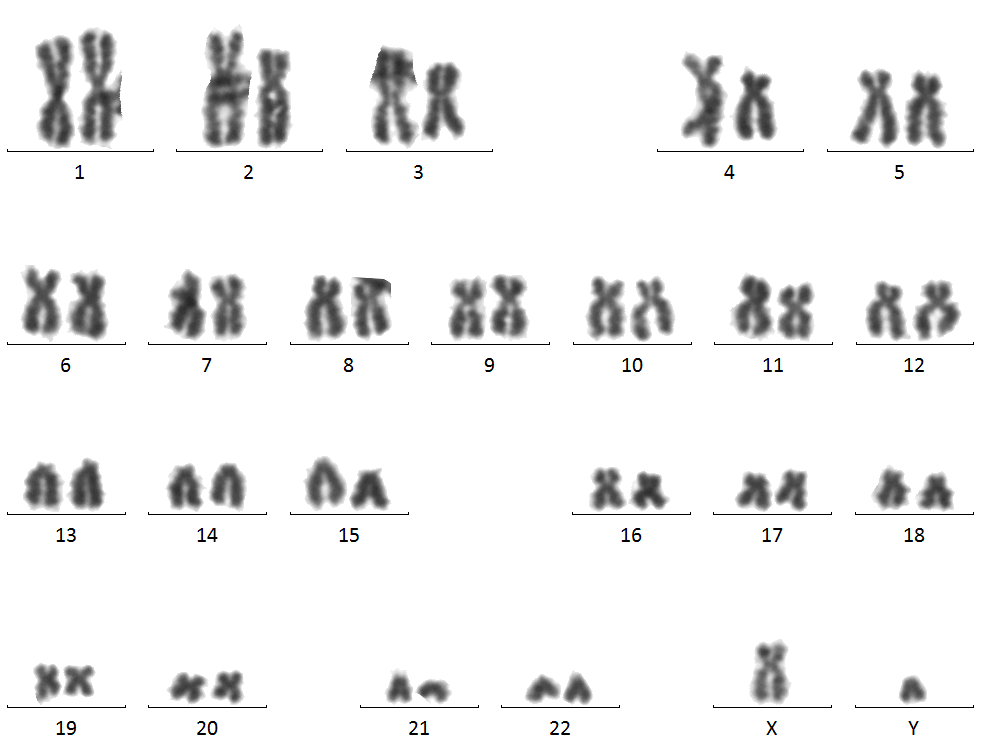
**

**DG75 control**

**A**

**DG75 DIV**

**B**

**
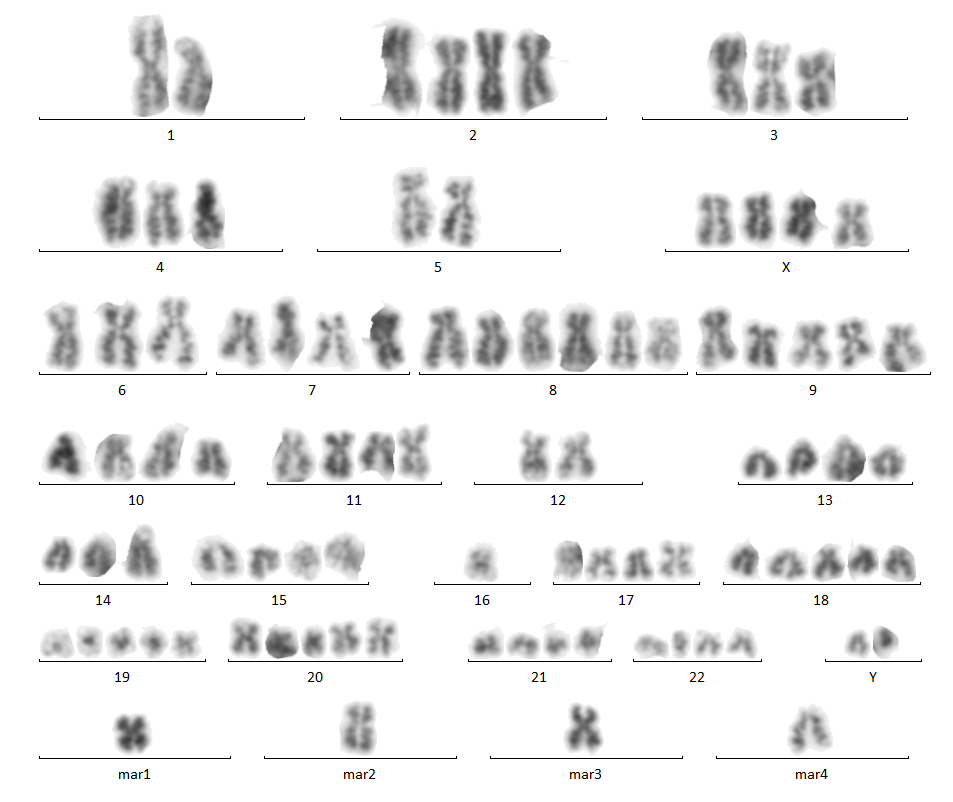
**

**C**

**Figure S1: DDR1 and COLVI expression in DLBCL**

A) Validation of the specificity of the DDR1 antibody. DG75 cells transfected with a DDR1α expression plasmid show the presence of strongly stained cells by IHC whereas no staining was observed in cells transfected with the empty control vector. B) Immunoblotting of the same cells. C) Co-expression of CD20 and DDR1 in DLBCL. A representative example of multiplex IF for CD20 (red) and DDR1 (green) showing co-expression in tumor cells. A blood vessel positive for DDR1 but not CD20 is arrowed in the merged image. This case is representative of six separate cases stained by multiplex IF. Original magnification x400. D) Expression of type VI collagen and DDR1 in DLBCL. Multiplex immunofluorescence (IF) shows that CD20-positive germinal center (GC) B cells of tonsil lack DDR1 expression, whereas the epithelium (EP) is positive for DDR1. Collagen VI was also found to be largely absent from normal GC. Data are representative of staining on three separate tonsils. Original magnification x400. E) IF of DLBCL shows that DDR1-expressing CD20-positive tumor cells were intimately associated with type VI collagen. This case was representative of a total of six separate cases stained by multiplex IF. Original magnification x400. F) Quantification of type VI collagen staining in DDR1-positive and DDR1-negative DLBCL. The difference between DDR1-positive and DDR1-negative cases was not significant (*p*=0.41).

**Figure S2: DDR1 expression in DLBCL cell lines**

A) Immunoblotting results of endogenous expression of DDR1 protein in DLBCL cell lines, detected by specific DDR1 antibody (MW=125kDa). β-tubulin confirmed equal loading of the sample. B) DDR1 mRNA expression in DLBCL cell lines shown by RT-qPCR. Data shown are representative of three independent experiments.

**Figure S3: Genes negatively correlated with DDR1 expression in DLBCL are enriched for mitotic spindle associated genes**

A) Selected significant GO terms from the ontology analysis of genes positively correlated with DDR1 expression in DLBCL included ‘collagen catabolic process’, ‘collagen metabolic process’ and ‘wound healing’ as well as ‘regulation of apoptosis’ and ‘cell migration’, reflecting known DDR1 functions. B) Selected significant GO terms from the ontology analysis of genes negatively correlated with DDR1 expression in DLBCL included ‘chromosome organization’, ‘mitotic sister chromatid segregation’, and ‘chromosome segregation’. Asterisks shows GO terms associated with mitotic spindle functions.

**Figure S4: DDR1 expression and SCNA frequency in Chapuy *et al***

Comparison of DDR1 expression across the different genetic subtypes defined by Chapuy *et al*. Higher expression was observed in the C2 subtype (defined by a high frequency of bi-allelic inactivation of *TP53* and genomic instability), compared with C1, C3 and C4 subtypes, although these differences were not significant (*p*=0.07, *p*=0.13, *p*=0.06, respectively).

**Figure S5: Regulation of lymphoma-associated genes by DDR1 in primary and transformed GC B cells** A) Confirmation of the over-expression of DDR1 in primary human GC B cells. Representative example of DDR1 expression in transfected GC B cells (Tonsil#T-24). B) RT-qPCR analysis of the relative expression of selected target genes reported as either up- or down-regulated in the RNAseq analysis of collagen-treated DDR1-transfected primary GC B cells compared to the same cells transfected with an empty vector. Shown are data from three separate tonsils performed in triplicate. C) Shows the differential expression of these DDR1 targets in control- or collagen-treated DDR1-expressing B cell lymphoma cell lines. Data are representative of three biological replicates.

**Figure S6: DDR1 and CENPE co-expression in tonsil and DLBCL**

A) Validation of CENPE antibody. Specificity of the CENPE antibody shown by immunoblotting following the siRNA-mediated knockdown of CENPE in DG75 cells. B) Multiplex IF for DDR1 and CENPE in tonsils and DLBCL. Upper panels show multiplex IF for expression of DDR1, CD20 and CENPE in normal tonsils. Germinal center (GC) B cells lack expression of DDR1 but express CENPE. Original magnification x400. Inset shows staining for DDR1 and CENPE in squamous epithelium of the tonsil (original magnification x600 oil immersion lens). Lower panels show a representative example of a DDR1-positive DLBCL lacking CENPE expression (original magnification x400). Inset shows a higher power image (original magnification x600 oil immersion lens) of the merged image from this case. The staining shown is representative of three separate tonsils and six separate DLBCL samples.

**Figure S7: Immunoblotting of DG75 cells transfected with a constitutively activated DDR1 receptor (DIV) or a mutant receptor (mDIV)**

Compared to cells transfected with DDR1 but not exposed to collagen, untreated cells expressing a constitutively active receptor (DIV) showed high levels of phospho-DDR1. Untreated cells expressing the mutant receptor (mDIV) showed lower, but detectable, levels of phospho-DDR1. Data shown are representative of three separate biological replicates.

**Figure S8: Karyotype of DG75 following DDR1 expression**

A) Representative karyotype of DDR1-negative DG75 cells after 29h in culture used as a control. B) Example of an abnormal karyotype seen in DG75 cells transfected with a constitutively active DDR1 gene (DIV) showing an increase in chromosome number (91 chromosomes). C) Table showing statistical analysis of chromosome counts for three replicates separately.
