# Supplementary material for: The Overexpression of Collagen Receptor DDR1 is Associated With Chromosome Instability and Aneuploidy in Diffuse Large B‐Cell Lymphoma
Source: J Cell Mol Med. 2025 May 22;29(10):e70318. doi: 10.1111/jcmm.70318 (PMC12096173; doi:10.1111/jcmm.70318)
Supplement: Supplementary file 2 — Tables S1–S5. [file JCMM-29-e70318-s001.docx]

**Table S1A: Antibodies used in IHC/IF and immunoblotting**

| **Antibody** | **Supplier** | **Clone** | **Species** | **WB dilution** | **IHC dilution and condition** | **IF dilution and condition** |
| --- | --- | --- | --- | --- | --- | --- |
| DDR1 (D1G6) XP(R) | Cell Signaling | 5583S | Rabbit | 1:1000 | 1:50; citrate buffer; incubation: 4^o^C, O/N | 1:200; citrate buffer; incubation: 4^o^C O/N |
| DDR1 (Tyr792) | Cell Signaling | 11994S | Rabbit | 1:1000 | - | - |
| Collagen VI | LsBio | LS-B696 | Rabbit | - | 1:200; citrate buffer; incubation: 4^o^C, O/N | 1:600; citrate buffer; incubation: RT, 1h |
| Cenpe | Sigma | HPA042294 | Rabbit | 1:1000 | 1:1800; citrate buffer; incubation: 4^o^C, O/N | 1:6000; (tissue), 1:400 (cytospin); citrate buffer; incubation: 4^o^C, O/N |
| CD20 | Dako | L26 | Mouse | - | Ready to use; citrate buffer; incubation: 1h RT | |
| β-actin (13E5) (HRP) | Cell Signaling | 5125 | Rabbit | 1:1000 | - | - |
| β-tubulin (9F3) (HRP) | Cell Signaling | 5146 | Rabbit | 1:1000 | - | - |
| Polyclonal Goat anti-Rabbit (HRP) | Dako | P0448 | Goat | 1:1000 | - | - |
| Polyclonal Goat anti-mouse (HRP) | Dako | P0447 | Goat | 1:3000 | - | - |
| ImmPRESS HRP reagent Universal Anti Mouse/ Rabbit IgG | Vector Laboratories Ltd. | MP-7500 | Horse | - | Ready to use; 30min, RT | - |
| Clean Blot IP Detection Reagent (HRP) | Thermo Fisher Scientific | 21230 | - | 1:500 | - | - |

**Table S1B: Fluorochromes used in IF**

| Fluorophore | Company | Dilution | Conditions |
| --- | --- | --- | --- |
| Cyanine 5 | Perkin Elmer | 1:400 | Diluted in amplification diluent (FP1135), part of Opal™ Fluorophore kit; Incubation for 6min |
| Cyanine 3 | Perkin Elmer | 1:200 | Diluted in amplification diluent (FP1135), part of Opal™ Fluorophore kit; Incubation for 10min |
| FITC | Perkin Elmer | 1:200 | Diluted in amplification diluent (FP1135), part of Opal™ Fluorophore kit; Incubation for 10min |
| DAPI | Life Technology | 1:1000 | Diluted in PBS; incubation for 5min |

**Table S1C: List of assays used for RT-qPCR**

| **Gene** | **Assay ID** |
| --- | --- |
| ADAM12 | Hs01106101_m1 |
| DDR1 | Hs01058433_g1 |
| KLHL15 | Hs00399541_m1 |
| SRSF4 | Hs00900675_m1 |
| GCSAM | Hs00381190_m1 |
| CENPE | Hs01068241_m1 |
| GAPDH | Hs02758991_g1 |
| β2M | Hs00187842_m1 |
| PGK1 | Hs99999906_m1 |

**Table S2: Analysis of DLBCL cases after immunohistochemical staining based on:** expression of DDR1 (0-negative, 1-positive), CENPE and COLVI (0-negative, 1-weak, lower than GC and cells of microenvironment, 2-positive, same intensity or more intense compare to GC and cells of microenvironment, 9-not done), patient age, type (1-GC type, 2-non-GC type, 9-unclassifiable) and stage of the disease, ECOG (Eastern Cooperative Oncology Group) and RIPI (Performance Score: Revised International Prognostic Index).

| Case number | DDR1 | CENPE | COL6 | Age | Type | Stage | ECOG | RIPI |
| --- | --- | --- | --- | --- | --- | --- | --- | --- |
| 6 | 1 | 0 | 9 | 71 | 2 | 2 | 2 | 2 |
| 7 | 1 | 9 | 9 | 67 | 2 | 2 | 2 | 2 |
| 8 | 0 | 9 | 9 | 46 | 2 | 3 | 3 | 3 |
| 18 | 1 | 9 | 9 | 75 | 1 | 1 | 1 | 3 |
| 25 | 0 | 1 | 9 | 65 | 2 | 4 | 0 | 3 |
| 26 | 0 | 0 | 9 | 59 | **9** | 3 | 0 | 2 |
| 28 | 1 | 1 | 0 | 64 | 2 | 4 | 0 | 3 |
| 29 | 1 | 0 | 9 | 68 | 2 | 2 | 0 | 2 |
| 34 | 0 | 0 | 9 | 90 | 1 | 2 | 0 | 2 |
| 35 | 0 | 1 | 9 | 67 | 1 | 2 | 0 | 2 |
| 38 | 0 | 1 | 9 | 58 | 2 | 1 | 0 | 1 |
| 39 | 0 | 0 | 0 | 76 | 1 | 2 | 0 | 2 |
| 49 | 0 | 1 | 9 | 57 | 2 | 1 | 0 | 2 |
| 53 | 0 | 1 | 1 | 71 | 2 | 2 | 1 | 2 |
| 55 | 0 | 1 | 1 | 30 | 1 | 4 | 1 | 2 |
| 65 | 0 | 1 | 9 | 78 | 2 | 4 | 0 | 3 |
| 77 | 0 | 9 | 9 | 56 | 2 | 1 | 0 | 1 |
| 100 | 0 | 0 | 1 | 49 | 1 | 2 | 0 | 2 |
| 102 | 1 | 9 | 9 | 86 | 2 | 1 | 0 | 1 |
| 108 | 0 | 9 | 9 | 77 | 2 | 1 | 0 | 2 |
| 122 | 1 | 1 | 9 | 75 | 1 | 1 | 0 | 2 |
| 127 | 1 | 1 | 9 | 70 | 2 | 3 | 1 | 3 |
| 129 | 0 | 9 | 9 | 86 | 1 | 1 | 0 | 2 |
| 131 | 0 | 0 | 9 | 57 | 1 | 4 | 0 | 2 |
| 132 | 0 | 0 | 9 | 74 | 2 | 3 | 0 | 3 |
| 133 | 1 | 9 | 9 | 60 | 1 | 1 | 0 | 1 |
| 134 | 0 | 1 | 1 | 67 | 2 | 2 | 0 | 2 |
| 137 | 0 | 9 | 9 | 61 | 2 | 4 | 0 | 3 |
| 138 | 0 | 9 | 9 | 92 | 1 | 1 | 0 | 2 |
| 139 | 1 | 0 | 9 | 56 | 2 | 4 | 3 | 2 |
| 141 | 0 | 9 | 9 | 63 | 1 | 3 | 0 | 3 |
| 143 | 1 | 0 | 9 | 76 | 2 | 2 | 0 | 2 |
| 146 | 0 | 9 | 9 | 30 | 1 | 1 | 0 | 1 |
| 149 | 0 | 9 | 9 | 81 | 2 | 3 | 4 | 3 |
| 150 | 1 | 9 | 9 | 61 | 2 | 1 | 0 | 1 |
| 152 | 0 | 9 | 9 | 78 | 1 | 4 | 0 | 3 |
| 154 | 0 | 9 | 9 | 64 | 1 | 1 | 0 | 2 |
| 155 | 0 | 9 | 9 | 64 | 2 | 1 | 0 | 2 |
| 156 | 0 | 9 | 9 | 81 | 1 | 4 | 2 | 3 |
| 164 | 1 | 0 | 9 | 90 | 2 | 1 | 0 | 2 |
| 166 | 0 | 9 | 9 | 68 | 1 | 2 | 1 | 2 |
| 167 | 0 | 9 | 9 | 61 | 1 | 4 | 1 | 2 |
| 168 | 0 | 9 | 9 | 84 | 2 | 4 | 1 | 2 |
| 173 | 0 | 0 | 1 | 59 | 1 | 2 | 0 | 1 |
| 174 | 1 | 1 | 1 | 71 | 1 | 2 | 0 | 1 |
| 175 | 0 | 9 | 9 | 76 | 2 | 2 | 9 | 2 |
| 177 | 0 | 9 | 9 | 65 | 1 | 3 | 0 | 3 |
| 178 | 1 | 1 | 1 | 36 | 1 | 1 | 0 | 1 |
| 179 | 0 | 0 | 1 | 64 | 1 | 3 | 0 | 2 |
| 181 | 1 | 0 | 9 | 45 | 2 | 3 | 1 | 2 |
| 182 | 1 | 1 | 9 | 24 | 1 | 3 | 1 | 2 |
| 183 | 1 | 9 | 9 | 60 | 2 | 4 | 1 | 3 |
| 185 | 1 | 0 | 9 | 65 | 1 | 3 | 0 | 2 |
| 189 | 0 | 9 | 9 | 61 | 2 | 4 | 0 | 3 |
| 190 | 1 | 0 | 1 | 41 | 2 | 3 | 0 | 2 |
| 191 | 0 | 9 | 9 | 78 | 1 | 4 | 1 | 3 |
| 196 | 1 | 9 | 9 | 67 | 2 | 3 | 1 | 3 |
| 197 | 0 | 9 | 9 | 48 | 1 | 2 | 1 | 1 |
| 199 | 0 | 9 | 9 | 68 | 1 | 4 | 0 | 3 |
| 208 | 0 | 9 | 9 | 75 | 1 | 1 | 0 | 2 |
| 211 | 1 | 0 | 9 | 85 | 2 | 2 | 0 | 2 |
| 212 | 0 | 9 | 9 | 70 | 1 | 1 | 0 | 2 |
| 215 | 0 | 9 | 9 | 71 | 1 | 4 | 0 | 2 |
| 220 | 1 | 0 | 1 | 51 | 1 | 4 | 0 | 3 |
| 221 | 0 | 9 | 9 | 46 | 1 | 1 | 0 | 2 |
| 226 | 0 | 1 | 1 | 60 | 2 | 2 | 1 | 2 |
| 227 | 0 | 9 | 9 | 70 | 2 | 4 | 1 | 3 |
| 228 | 1 | 0 | 1 | 76 | 2 | 1 | 0 | 2 |
| 234 | 1 | 0 | 1 | 45 | 1 | 2 | 0 | 2 |
| 239 | 1 | 0 | 9 | 44 | 2 | 2 | 3 | 2 |
| 240 | 1 | 0 | 9 | 82 | 2 | 4 | 1 | 2 |
| 241 | 1 | 0 | 9 | 74 | 1 | 2 | 0 | 2 |
| 243 | 1 | 0 | 9 | 56 | 2 | 1 | 0 | 2 |
| 247 | 0 | 1 | 1 | 71 | 2 | 3 | 0 | 3 |
| 251 | 0 | 9 | 9 | 76 | 2 | 1 | 0 | 2 |
| 252 | 0 | 0 | 1 | 66 | 1 | 3 | 0 | 3 |
| 258 | 0 | 0 | 0 | 61 | 1 | 3 | 0 | 3 |
| 260 | 1 | 1 | 1 | 81 | 2 | 2 | 0 | 2 |
| 261 | 1 | 0 | 1 | 83 | 1 | 3 | 0 | 3 |
| 262 | 1 | 1 | 9 | 51 | 1 | 1 | 0 | 1 |
| 263 | 1 | 0 | 9 | 81 | 2 | 2 | 0 | 2 |
| 265 | 0 | 9 | 9 | 62 | 1 | 1 | 0 | 2 |
| 267 | 1 | 0 | 1 | 71 | 2 | 4 | 0 | 3 |
| 268 | 0 | 0 | 1 | 77 | 2 | 4 | 1 | 3 |
| 275 | 0 | 9 | 9 | 51 | 2 | 3 | 1 | 1 |
| 294 | 1 | 0 | 9 | 60 | 2 | 3 | 0 | 2 |
| 299 | 1 | 0 | 1 | 75 | 1 | 1 | 0 | 2 |
| 304 | 1 | 1 | 9 | 59 | 2 | 4 | 0 | 2 |
| 305 | 0 | 9 | 9 | 71 | 2 | 4 | 2 | 2 |
| 306 | 1 | 9 | 9 | 75 | 2 | 2 | 0 | 2 |

**Table S3: List of known collagen genes**

Source: http://www.genenames.org/genefamilies/COLLAGEN

| Gene name | Approved Name | Previous Symbols | Synonyms | Chromosome | Accession Numbers | RefSeq IDs |
| --- | --- | --- | --- | --- | --- | --- |
| COL1A1 | collagen type I alpha 1 |  | OI4 | 17q21.33 | Z74615 |  |
| COL1A2 | collagen type I alpha 2 | OI4 |  | 7q21.3 | Z74616 | NM_000089 |
| COL2A1 | collagen type II alpha 1 | SEDC, AOM | STL1 | 12q12-q13.2 | X16468 | NM_001844 |
| COL3A1 | collagen type III alpha 1 | EDS4A |  | 2q32.2 | X15332 | NM_000090 |
| COL4A1 | collagen type IV alpha 1 |  |  | 13q34 | J04217 |  |
| COL4A2 | collagen type IV alpha 2 |  | FLJ22259, DKFZp686I14213 | 13q34 | AK025912 | NM_001846 |
| COL4A3 | collagen type IV alpha 3 |  |  | 2q36-q37 |  | NM_000091 |
| COL4A4 | collagen type IV alpha 4 |  | CA44 | 2q35-q37 |  | NM_000092 |
| COL4A5 | collagen type IV alpha 5 | ASLN, ATS |  | Xq22 | M90464 |  |
| COL4A6 | collagen type IV alpha 6 |  |  | Xq22 | U04845 |  |
| COL5A1 | collagen type V alpha 1 |  |  | 9q34.2-q34.3 | D90279 | NM_000093 |
| COL5A2 | collagen type V alpha 2 |  |  | 2q14-q32 | Y14690 | NM_000393 |
| COL5A3 | collagen type V alpha 3 |  |  | 19p13.2 | AF177941 | NM_015719 |
| COL6A1 | collagen type VI alpha 1 |  |  | 21q22.3 | M20776 | NM_001848 |
| COL6A2 | collagen type VI alpha 2 |  |  | 21q22.3 | M20777 |  |
| COL6A3 | collagen type VI alpha 3 |  |  | 2q37 | X52022 | NM_004369 |
| COL6A4P1 | collagen type VI alpha 4 pseudogene 1 | DVWA | VWA6, DIVA, COL6A4, COL6A4P | 3p25.1 | AB299979 | NR_027927 |
| COL6A4P2 | collagen type VI alpha 4 pseudogene 2 |  | COL6A4 | 3q22.1 | BF593933 | NR_027898 |
| COL6A5 | collagen type VI alpha 5 | COL29A1 | FLJ35880, VWA4 | 3q21.3 | AK093199 | NM_153264 |
| COL6A6 | collagen type VI alpha 6 |  |  | 3q22.1 | AL713792 | NM_001102608 |
| COL7A1 | collagen type VII alpha 1 | EBDCT, EBD1, EBR1 |  | 3p21.1 | L02870 | NM_000094 |
| COL8A1 | collagen type VIII alpha 1 | C3orf7 | MGC9568 | 3q11.1-q13.2 | AF170702 | NM_001850 |
| COL8A2 | collagen type VIII alpha 2 | FECD | PPCD, FECD1, PPCD2 | 1p34.2-p32.3 | M60832 | NM_005202 |
| COL9A1 | collagen type IX alpha 1 |  |  | 6q13 |  |  |
| COL9A2 | collagen type IX alpha 2 | EDM2 | MED | 1p33-p32 | M95610 | NM_001852 |
| COL9A3 | collagen type IX alpha 3 |  | IDD, MED, EDM3, FLJ90759, DJ885L7.4.1 | 20q13.3 | AK075240 | NM_001853 |
| COL10A1 | collagen type X alpha 1 |  |  | 6q21-q22 |  |  |
| COL11A1 | collagen type XI alpha 1 | COLL6 | STL2, CO11A1 | 1p21 | J04177 | NM_080630 |
| COL11A2 | collagen type XI alpha 2 | DFNA13, DFNB53 | HKE5 | 6p21.3 | U32169 |  |
| COL12A1 | collagen type XII alpha 1 | COL12A1L |  | 6q12-q13 | U73779 | NM_004370 |
| COL13A1 | collagen type XIII alpha 1 |  |  | 10q22 | AJ293624 | NM_005203 |
| COL14A1 | collagen type XIV alpha 1 | UND |  | 8q23 |  | NM_021110 |
| COL15A1 | collagen type XV alpha 1 |  |  | 9q21-q22 | L25286 | NM_001855 |
| COL16A1 | collagen type XVI alpha 1 |  |  | 1p35-p34 | M92642 | NM_001856 |
| COL17A1 | collagen type XVII alpha 1 | BPAG2 | BP180 | 10q24.3 | M91669 | NM_130778, NM_000494 |
| COL18A1 | collagen type XVIII alpha 1 | KNO | KS, KNO1 | 21q22.3 |  |  |
| COL19A1 | collagen type XIX alpha 1 |  |  | 6q12-q13 |  |  |
| COL20A1 | collagen type XX alpha 1 |  | KIAA1510 | 20q13.33 | BC043183 | NM_020882 |
| COL21A1 | collagen type XXI alpha 1 |  |  | 6p12.3-p11.2 | AF330693 |  |
| COL22A1 | collagen type XXII alpha 1 |  |  | 8q24.3 | AF406780 | XM_291257 |
| COL23A1 | collagen type XXIII alpha 1 |  | DKFZp434K0621 | 5q35.3 | AL137461 | NM_173465 |
| COL24A1 | collagen type XXIV alpha 1 |  |  | 1p22.3-p22.2 | AF410793 | NM_152890 |
| COL25A1 | collagen type XXV alpha 1 |  |  | 4q25 | AF293340 | NM_032518 |
| COL26A1 | collagen type XXVI alpha 1 | EMID2 | Emu2, EMI6 | 7q22.1 | AJ416091 | NM_133457 |
| COL27A1 | collagen type XXVII alpha 1 |  | KIAA1870, MGC11337, FLJ11895 | 9q33.1 | AB058773 | NM_032888 |
| COL28A1 | collagen type XXVIII alpha 1 |  |  | 7p21.3 | AJ890451 | NM_001037763 |

**Table S4A: A list of 513 ‘mitotic spindle associated’ genes (‘GO:0031577’)**

| ANAPC1 | ACTR2 | AHCTF1 | AKT1 | ACOT13 | ARL8A | ALMS1 |
| --- | --- | --- | --- | --- | --- | --- |
| ANAPC10 | ACTR3 | AKAP8L | ANXA11 | AGBL5 | ASPM | ANAPC15 |
| ANAPC11 | AGO4 | ANKRD53 | APITD1 | ARHGEF2 | CCSAP | ANKLE1 |
| ANAPC2 | AKAP8 | B9D2 | APP | ARL2BP | CENPV | APC |
| ANAPC4 | ATRX | BRIP1 | ARL3 | ARL8B | CEP128 | BRCA1 |
| ANAPC5 | AXIN2 | CCNB1IP1 | BIRC2 | AUNIP | CEP170 | BRCA2 |
| ANAPC7 | BAG6 | CDCA8 | BIRC3 | BIRC6 | CEP57L1 | CASC5 |
| ATAT1 | BECN1 | CENPH | CALM1 | BIRC7 | CHAMP1 | CCDC155 |
| ATM | CCNE1 | CENPK | CAPG | BRCC3 | CKAP2L | CDCA5 |
| AURKA | CCNE2 | CENPM | CAPN6 | CBX1 | DNAAF1 | CDK5RAP2 |
| AURKAIP1 | CDC14A | CENPN | CDC25B | CBX3 | DYNLL1 | CENPF |
| AURKB | CDC42 | CENPO | CDK1 | CEP162 | FAM110C | CENPL |
| AURKC | CDC6 | CENPQ | CENPP | CEP85 | FRY | CHMP4B |
| BCL2L11 | CENPA | CENPT | CEP104 | CIAO1 | GEN1 | CHMP4C |
| BIRC5 | CENPC | CENPU | CEP19 | CKAP2 | GSG2 | CHMP7 |
| BIRC8 | CENPI | CEP89 | CEP350 | CTDP1 | HDAC3 | CLTCL1 |
| BUB1 | CEP44 | CHMP1B | CETN1 | DCTN3 | HEPACAM2 | CSNK2A1 |
| BUB1B | CEP57 | CHMP2A | CLTA | DIDO1 | ITGB3BP | CSNK2A2 |
| BUB3 | CEP63 | CHMP2B | CLTC | EML2 | KATNB1 | CTCF |
| CCNA1 | CHMP1A | CHMP4A | CRMP1 | FAM96B | KIF20A | DIS3L2 |
| CCNA2 | CKAP5 | CHMP5 | CSNK1D | GPSM2 | KIF3B | DMC1 |
| CCNB1 | CLIP1 | CHMP6 | CTTN | HAUS1 | KIF4B | DUSP1 |
| CCNB2 | CTNNB1 | DSCC1 | CYLD | HAUS2 | KLHL21 | DYNC1LI1 |
| CCNB3 | CUL3 | DSN1 | DCTN1 | HAUS3 | KLHL22 | DYNLT1 |
| CDC16 | DDX11 | EME2 | DIAPH1 | HAUS4 | LCMT1 | EME1 |
| CDC20 | DLGAP5 | ERCC6L | DYNC1I1 | HAUS5 | LEMD2 | ESCO1 |
| CDC23 | ECT2 | FAM83D | E4F1 | HAUS6 | LEMD3 | EVI5 |
| CDC26 | ERCC4 | FANCM | EFHC1 | HAUS7 | MAEA | HFM1 |
| CDC27 | FAM110A | FMN2 | EML1 | INVS | MAU2 | HORMAD2 |
| CDC34 | FAM175B | HDAC8 | ERCC2 | JTB | MEIOB | IKBKG |
| CDKN1A | FANCD2 | HORMAD1 | FBF1 | KATNA1 | MZT1 | KIF18B |
| CENPE | FEN1 | INO80 | GOLGA2 | KIF15 | MZT2A | KIF2C |
| CEP250 | GEM | KBTBD8 | HSPA2 | KIF20B | NCAPD3 | KNSTRN |
| CEP95 | H2AFY | KIF18A | KAT2A | KIF3A | NCAPH | M1AP |
| CLASP1 | INCENP | KIF2B | KIF11 | KIFAP3 | NDRG1 | MAPRE1 |
| CLASP2 | KIF14 | KIF4A | LRMP | KLHL42 | NEDD1 | MEI1 |
| CSE1L | KIF22 | MAEL | MAK | LATS2 | NEIL2 | MRE11A |
| CSPP1 | KIF23 | MAP10 | MAP2K5 | MAD2L1BP | NEK7 | NEK6 |
| ESPL1 | KIF25 | MCMBP | MAP4 | MAP1S | NSMCE2 | NUDC |
| FBXO5 | KIF2A | MIS12 | MAPK1 | MAP7D1 | NUBP2 | P3H4 |
| FBXW7 | KIFC1 | MLH3 | MAPK14 | MAP7D3 | NUDCD2 | PAPD7 |
| FZR1 | KPNB1 | MUS81 | MEI4 | MMS19 | NUP160 | PDS5B |
| HAUS8 | LATS1 | NAA50 | MEIKIN | MTCL1 | PCID2 | PHB2 |
| HSPB1 | MLH1 | NCAPG | MID1 | MTUS1 | PDS5A | PHF13 |
| KNTC1 | MOS | NCOR1 | MYH10 | NIN | POC1B | PIBF1 |
| MAD1L1 | MSH4 | NDC1 | MYH9 | NLRC4 | POGZ | PKP4 |
| MAD2L1 | MSH5 | NDE1 | NEDD9 | NSUN2 | PSMG2 | PLK1 |
| MAD2L2 | NCAPD2 | NDEL1 | NPM1 | NUP62 | RASSF10 | PMF1 |
| MAP9 | NUP98 | NIPBL | NR3C1 | OR2A4 | RMDN2 | PPP1CA |
| MZT2B | PAFAH1B1 | NSL1 | NUMA1 | PLEKHG6 | RNF212 | PPP1R2 |
| NDC80 | PPP1CC | NUF2 | NUP43 | POC1A | RRS1 | PTP4A1 |
| NEDD8 | PPP2R1A | NUP107 | ODF2 | PPP2R3C | SEPT12 | PTTG2 |
| NEK2 | PRC1 | NUP37 | PARP4 | PRPF19 | SKA1 | RAD21 |
| NUP133 | RAB11A | NUP85 | PIN4 | RASSF1 | SKA3 | RNF2 |
| PRCC | RAD51C | NUSAP1 | PKD2 | RGS14 | SPDL1 | SIRT2 |
| PRPF4B | RANBP2 | PINX1 | PKHD1 | RIF1 | SPO11 | SMC1B |
| PTTG1 | RANGAP1 | PSRC1 | POLB | RMDN1 | STRA8 | SMC2 |
| RAE1 | RB1 | RAB11FIP4 | PPP2CA | RMDN3 | STX1B | SPAG5 |
| RAN | REC8 | RACGAP1 | PPP2CB | SAC3D1 | SUN1 | SPC24 |
| RCC1 | RPS27 | RCC2 | RAD21L1 | SBDS | SYCE2 | SPICE1 |
| SGOL2 | SEC13 | SEH1L | RNF212B | SEPT6 | TADA3 | STAG3 |
| SMC1A | SFPQ | SLF1 | RPS3 | SHCBP1 | TEX14 | SYCE1 |
| SMC3 | SHCBP1L | SLF2 | RPS6KA2 | SPAG8 | TOPORS | SYCP2 |
| STAG1 | SMC4 | SLX4 | RSPH1 | SPECC1L | TPR | TBL1X |
| STAG2 | SMC5 | SPATA22 | SEPT2 | SPIN1 | TTC23L | TUBG1 |
| TACC1 | SYCP1 | SPC25 | SEPT7 | TBCCD1 | TUBB8 | UBE2B |
| TACC2 | TERF1 | STRA13 | SKA2 | TBL1XR1 | TUBGCP3 | UBE2C |
| TACC3 | TNKS | TAOK1 | SLC25A5 | TOPBP1 | UNC119 | UBE2N |
| TPX2 | TOP2A | TEX11 | SNCG | TTC28 | USP44 | UMOD |
| TTK | TOP2B | TEX12 | SPAST | TUBG2 | VRK1 |  |
| UBD | TRIP13 | TEX15 | SYCE1L | TUBGCP2 | WAPL |  |
| ZW10 | TTN | TRAPPC12 | SYCE3 | TUBGCP4 | WDR62 |  |
| ZWILCH | VPS4B | VPS4A | TUBGCP6 | TUBGCP5 | XRCC3 |  |
| ZWINT | XPO1 | WDR73 | XIAP | YEATS2 | ZNF207 |  |

**Table S4B: 103 genes associated with the mitotic spindle checkpoint (‘GO:0031577’)**

| ANAPC1 | CASC5 | CSNK2A1 | NEK2 | STAG1 |
| --- | --- | --- | --- | --- |
| ANAPC10 | CCNA1 | CSNK2A2 | NUP133 | STAG2 |
| ANAPC11 | CCNA2 | DUSP1 | PCID2 | TACC1 |
| ANAPC15 | CCNB1 | DYNC1LI1 | PLK1 | TACC2 |
| ANAPC2 | CCNB2 | ESPL1 | PPP1CA | TACC3 |
| ANAPC4 | CCNB3 | FBXO5 | PPP1R2 | TEX14 |
| ANAPC5 | CDC16 | FBXW7 | PRCC | TPR |
| ANAPC7 | CDC2 | FZR1 | PRPF4B | TPX2 |
| APC | CDC20 | GEN1 | PSMG2 | TTK |
| ATM | CDC23 | GSG2 | PTTG1 | UBD |
| AURKA | CDC26 | HSPB1 | RAD21 | UBE1C |
| AURKAIP1 | CDC27 | KLHL22 | RAE1 | UBE2B |
| AURKB | CDC34 | KNTC1 | RAN | UBE2N |
| AURKC | CDK5RAP2 | LCMT1 | RASSF1A | USP44 |
| BCL2L11 | CDKN1A | MAD1L1 | RCC1 | XRCC3 |
| BIRC5 | CENPE | MAD2L1 | RNF2 | ZNF207 |
| BRCA1 | CENPF | MAD2L2 | SGOL2 | ZW10 |
| BRCA2 | CEP250 | MRE11A | SMC1A | ZWILCH |
| BUB1 | CLASP1 | NDC80 | SMC1B | ZWINT |
| BUB1B | CLASP2 | NDRG1 | SMC3 |  |
| BUB3 | CSE1L | NEDD8 | SPDL1 |  |

**Table S5A: Genes upregulated after collagen stimulation of primary GC B cells transfected with DDR1 (*p*<0.05; fold change>1.5)**

| DDR1 | GCDH | TMEM241 | VSNL1 | MRC2 |
| --- | --- | --- | --- | --- |
| GNAT1 | PPP2R4 | LRRC14 | BAMBI | DCHS2 |
| SYNGR2 | KCNMA1 | ZNF668 | SERHL | SNORA70D |
| PKM | ORC5 | MIR4712 | RAB9B | NEUROG3 |
| TRPM7 | RHOT1 | LINC01393 | TST | ASIC4 |
| PTPRS | ZNF850 | MAPKAPK3 | SYNPO | LOC442028 |
| MFHAS1 | MARK4 | ZSWIM5 | MID1 | PHLDB1 |
| EIF3E | JUP | LOC101927190 | VAMP5 | TGFA |
| ATP2A3 | CABLES2 | EPHB2 | PTGES2-AS1 | MRAP2 |
| CYB561A3 | PLN | AIRN | ZNF702P | EXOC3L1 |
| PHF19 | SLC25A1 | PRKCA-AS1 | FOXD2-AS1 | FAM215A |
| HSPB1 | RAB24 | WNK3 | SOX10 | CNTN6 |
| KCNC3 | IFI44 | SYTL4 | ST3GAL4-AS1 | RERG |
| TP53I11 | PGRMC1 | ARHGEF19 | C15orf52 | MAGEE1 |
| SEPT9 | RIC8A | CENPB | KIRREL3 | SMIM1 |
| ATG7 | MC1R | FBP1 | WIPF3 | RPRML |
| KMT2B | RAP2C-AS1 | RHCG | LKAAEAR1 | CLRN1-AS1 |
| CECR7 | C1orf198 | RASA4CP | DNAH5 | ALOX12B |
| C7orf49 | MRPS34 | LAMA3 | PCDHB12 | SYN3 |
| OXCT1 | MRI1 | TUNAR | PTGES3L | C9orf57 |
| PRMT2 | DTHD1 | STX16-NPEPL1 | GNRH2 | LOC101928008 |
| PKN1 | MATN2 | NR2F6 | SNORD128 | FLT3LG |
| GMIP | CCDC17 | SCN8A | TRPM4 | SNORA70C |
| RPTOR | LOXL4 | HIST3H2A | MIR548U | CTC-436P18.1 |
| SNX8 | FBXL15 | FKSG29 | PRB1 | KIAA1656 |
| PKIG | ANKRD50 | DPP9-AS1 | CTSW | SPRNP1 |
| KLHL6-AS1 | RUSC1 | AKAP3 | GLYATL1 | IFNA21 |
| TRAPPC9 | OBSCN | STX17-AS1 | SNORD72 | CDK20 |
| SIRPA | LDLR | LINC00649 | SCARNA26B | COX7A1 |
| PSD4 | MYO15B | PDE4C | LOC283683 | PRR22 |
| TMEM68 | INPP4B | PPIL6 | FAM174B | MYO3A |
| DCUN1D2 | ARFRP1 | LOC101927575 | HSF2BP | HCRTR1 |
| CSK | CARNS1 | ATP1A3 | TERT | ZP2 |
| SCIMP | SMPD3 | SNORA23 | LBX2 | RAPSN |
| RNF32 | RFX3-AS1 | RAB34 | TMIGD3 | CCDC61 |
| BTF3L4 | MROH1 | PPP1R2P9 | UNC5B | FBF1 |
| PHLDA1 | ADAM12 | ITPRIPL1 | PKNOX2 | LINC00704 |
| PTPRCAP | HEXDC | LOC101927322 | MIR3675 | RARG |
| DAGLB | TACR1 | GPATCH3 | ADH4 | PIWIL3 |
| ABCC5 | ERMAP | LINC00562 | LOC100996324 | WARS2-IT1 |
| CARF | ALKBH2 | GP1BA | LVRN | LDOC1 |
| GRWD1 | FMO5 | PRR7-AS1 | TRPM5 | CA11 |
| FTX | TMTC2 | C1orf216 | TMEM59L | PRDM7 |
| ELOVL1 | KLHL35 | WNT3 | C7orf71 | SORCS2 |
| TYMP | CLIC5 | PTGDR2 | SAG | LYVE1 |
| TMEM185B | LHFPL3-AS2 | LINC01279 | SYT16 | CREB3L1 |
| MAF1 | OCEL1 | SIGLEC5 | FAM188B | LAMC2 |
| GALNT14 | ZNF784 | OR2T11 | MLC1 | PROSER2-AS1 |
| ABAT | ZNRD1-AS1 | AGAP3 | LINC00901 | PIK3IP1-AS1 |
| RNPEPL1 | FAM189B | TIMP2 | LINC01221 | FOLR2 |
| CILP | TMEM184A | HEYL | KCNC4-AS1 | MMP9 |
| LINC01511 | LOC339059 | CSF3R | FIRRE | WFIKKN1 |
| USP46-AS1 | BAALC-AS2 | DDX4 | BTNL10 | MIR5189 |
| SLC39A2 | SIM2 | LPO | SLC7A10 | CSPG4 |
| TCEB3B | LINC01586 | LOC100289673 | LOC285043 | RPL3L |
| STRC | KCNK10 | LOC100505625 | CD34 | LOC101928751 |
| DISC1 | USP27X-AS1 | HRG | OSGIN1 | C20orf203 |
| GRIN2D | KLF15 | MIR623 | CYP7A1 | C11orf42 |
| EMX1 | MCF2L-AS1 | OR5P2 | HP09025 | HS3ST1 |
| C16orf90 | MIR302B | FGD5P1 | ZFPM2-AS1 | SH2D7 |
| IQSEC2 | WISP2 | WNT8A | LILRA5 | FAM109A |
| SMARCD3 | ABCA3 | OR52H1 | SCARNA22 | DRAXIN |
| C2-AS1 | FGFBP1 | GRB7 | LINC00705 | ART5 |
| TMEM51 | NOXA1 | SYDE2 | C19orf57 | TTLL10 |
| ADAMTS20 | FOXL1 | LOC100507506 | DEFA8P | LINC00595 |
| DRP2 | LOC101927914 | FAM46B | LOC101929241 | LOC389332 |
| LOC375196 | MOCOS | LINC00460 | GPR83 | IL25 |
| DNM1P35 | ESYT3 | CHRND | SV2B | CYP3A7-CYP3A51P |
| LOC286370 | HHIP-AS1 | B4GALNT2 | CYP26A1 | AVPR2 |
| KRTAP11-1 | MGC45922 | PDZD7 | FAM167B | TARM1 |
| MIR365A | RBM20 | SLC5A12 | GRK7 | MLNR |
| TMPRSS11F | XYLB | RAPGEF4-AS1 | MIR5739 | CHST3 |
| CKM | LOC101927666 | LINC00461 | MIR8055 | ADCY6 |
| TSPAN2 | FAM181A | TNNI2 | mar.04 |  |
| CYP46A1 | ZNF423 | EVA1A | TDRD10 |  |
| MORN3 | FPR3 | DIRAS3 | RBBP8NL |  |
| MPL | NUPR1 | MYOC | TBX2-AS1 |  |
| RNF180 | RHOD | PCDHB19P | KRTAP7-1 |  |
| RNY5 | AZIN2 | FSTL3 | RNF112 |  |
| NALCN-AS1 | LOC100507406 | CLDN2 | F2RL1 |  |
| LOC101928514 | HR | GPR1-AS | SLC9C2 |  |
| COMMD3-BMI1 | LOC400655 | LOC101929128 | SUSD2 |  |

**Table S5B: Genes downregulated after collagen stimulation of primary GC B cells transfected with DDR1 (*p*<0.05; fold change>1.5)**

| PLK5 | KCTD21-AS1 | TMEM158 | LOC100133920 | DNAH6 |
| --- | --- | --- | --- | --- |
| HTR3D | XLOC_009911 | MIR4651 | SDC2 | ALDH8A1 |
| MCCD1 | NEK5 | TAS2R3 | SPARC | ZNF695 |
| PLXDC2 | SP7 | LOC100505878 | MCHR1 | ZNF329 |
| STARD13 | CDHR4 | LINC01185 | F13B | CDH1 |
| WFDC5 | MLIP | PREX2 | AMN | LHFP |
| CHIA | COX4I2 | XLOC_008559 | CEACAM22P | CLIP3 |
| ESPNL | CCDC87 | MIR5696 | SERPINA11 | LOC101927021 |
| FLT4 | THSD1 | KLK4 | ART4 | LOC101927438 |
| MIR4786 | CCL21 | HRAT56 | GRIA4 | DNAJC3-AS1 |
| SAXO1 | ZMIZ1-AS1 | HIST1H4G | RIN1 | RELL1 |
| CYP4F2 | SHISA9 | SIX5 | ACADS | MUC20 |
| LYPD3 | ZBTB47 | MIR10A | PLEKHB1 | BAD |
| SLC40A1 | CCL20 | TCEAL5 | LOC101927157 | AK1 |
| EDN3 | EGFLAM | IL20 | GPSM1 | ADCY10 |
| MIR140 | NLGN2 | ZFP57 | ADM5 | CYP39A1 |
| CHRDL2 | ADAM33 | LINC00643 | LOC102724919 | CASP10 |
| GPR84 | LINC00239 | LOC101928855 | MIR197 | MTOR-AS1 |
| FASLG | C20orf78 | LCA10 | GIPR | SLC25A11 |
| RD3L | HIST1H4L | PCSK4 | GSDMA | TBC1D19 |
| MIR7-3HG | MIR5003 | ATP13A5 | SHOX2 | PINK1-AS |
| OXCT1-AS1 | TMEM169 | IGFBP6 | NLRP6 | NPRL2 |
| RNF148 | LOC101927623 | LOC100128239 | PXN-AS1 | NAGPA |
| CLDN4 | DOK5 | FOXG1 | ADSSL1 | BORCS8 |
| SOCS2-AS1 | ITGA9 | LINC01093 | TTC39A | NAPSA |
| MEIS3 | IDO2 | HIST1H1T | C4orf26 | CCNF |
| MOCS1 | SYT7 | C2orf57 | SKOR2 | MLKL |
| PLA2G2D | ATP6V0D2 | HS6ST2 | TUBB4A | LINC00094 |
| NTRK1 | PDGFB | NYNRIN | MIR4681 | CMYA5 |
| PRSS48 | FOXL2 | PLA2G4E | FUOM | RAD54L |
| SSC4D | BLACE | HOMER3 | SUGCT | ERF |
| UBE2E1-AS1 | XIRP2 | CCDC40 | LINC00184 | TSPAN18 |
| FER1L5 | PLA2G4A | LOC102724009 | DDN | SERGEF |
| LOC102723354 | SATL1 | MIR3605 | SIX4 | SLC25A28 |
| GATA6 | LOC100130370 | EVPL | SNORD83B | CADM1 |
| APBA1 | HIST1H3G | BSX | LOC102723703 | PAQR8 |
| ATP4A | C1R | FLJ46906 | MTUS2-AS1 | MYO1A |
| LINC00442 | NBEAP1 | NLRP5 | TNFRSF1A | ALOX5AP |
| LEP | TBX5 | UPB1 | SPRY4 | TSIX |
| CEACAM20 | ZMYND15 | LINC00937 | RIBC1 | MIR4435-2HG |
| CD300C | TMEM119 | RAB19 | IL22RA1 | ZNF473 |
| CNKSR3 | LOC100996286 | HIST2H3D | SCML1 | GLRX5 |
| SLC1A6 | ENTHD1 | DDX11L2 | DTWD2 | ABHD5 |
| C11orf70 | PRRX2 | ZNF462 | GGT7 | ADCK3 |
| AP1M2 | HKDC1 | MIR23A | LRRC46 | NAA40 |
| LOC101928911 | HOGA1 | ATCAY | SLC25A35 | GET4 |
| KCNT1 | ANKK1 | DUSP5P1 | MOSPD3 | ZNF724P |
| TSPAN10 | TRPM8 | SNORA51 | HMGN2P46 | AACS |
| ALS2 | CENPE | CECR1 | VRK3 | LMNB2 |
| GDAP1 | SNHG15 | CREG1 | ERN1 | MYB |
| GCSAM | GPATCH4 | ANAPC7 | USP11 | SRSF4 |
| ATG9A | KLHL15 | MXD1 | NR4A2 | PRDM1 |

**COLVI**

**merged**

**CD20**

**DDR1**

**CD20**
