# Supplementary material for: The Overexpression of Collagen Receptor DDR1 is Associated With Chromosome Instability and Aneuploidy in Diffuse Large B‐Cell Lymphoma
Source: J Cell Mol Med. 2025 May 22;29(10):e70318. doi: 10.1111/jcmm.70318 (PMC12096173; doi:10.1111/jcmm.70318)
Supplement: Supplementary file 3 — Data S1. [file JCMM-29-e70318-s002.docx]

**Supplementary Materials and Methods**

***Digital semi-automated quantitative scoring***

Vectra system version 3.0.3 software (PerkinElmer, Seer Green, UK) was used for multispectral imaging. Following a whole slide scan, Phenochart 1.0.4 (PerkinElmer) was used for manual selection of representative areas of interest. 3 HPF (20x) were used for quantification. Scanning of these fields using the high-power protocol was applied on selected areas. Multispectral images were acquired using Inform Tissue Finder (Version 2.4.0, PerkinElmer) software. The segmentation step was used to detect cells based on DAPI expression in cell nucleus, followed by cell phenotyping. Positivity of markers was based on intensity of the individual marker for individual fluorophores (Table S1B) following manual selection of the positive threshold intensity. Cells were classified into four groups: DDR1+CENPE-; DDR1+CENPE+, DDR1-CENPE-, DDR1-CENPE+.

***Global gene expression analysis***

Tonsils were minced and mononuclear cells isolated by Ficoll-Isopaque centrifugation. CD10-positive GC B cells were isolated by magnetic separation at 4°C with anti-CD10-Phycoerythrin (PE) (eBioscience, San Diego, CA, USA), anti-PE microbeads and LS columns (both Miltenyi Biotec Ltd., Surrey, UK) following the protocol of the manufacturer. Transfection was performed by nucleofection using the Cell Line Nucleofector Kit B (Amaxa, Cologne, Germany). Cell pellets from sorted, transfected, GC B cells were lysed in 100µl of RLT buffer (QIAGEN Ltd, Manchester, UK) supplemented with 1µl of 14.3M β-mercaptoethanol (Sigma-Aldrich, Dorset, UK) and 1µl of N-carrier (AmpTec, Hamburg, Germany). RNA extraction was performed as per manufacturer’s protocol for the QIAGEN RNeasy micro kit (QIAGEN Ltd). The quality and concentration of RNA was tested using the Bioanalyser 2100 (Agilent Technologies, Stockport, UK) with the Agilent RNA 6000 Pico Kit (Agilent Technologies). Amplification of cDNA was performed using NuGEN Ovation® RNA-Seq system V2 kit (NuGEN Ltd, Leek, The Netherlands), according to the manufacturer’s protocol. For RNAseq, TruSeq Nano libraries were generated from amplified cDNA and sequenced on HISeq4000 for Illumina paired-end RNA sequencing (125 base pairs; Edinburgh Genomics, Edinburgh, UK). Quality control of raw sequencing files was done using FastQC software. FastQC tool was used to examine read base quality, sequence quality, length, over-represented reads and extent of sequence duplication in the fastq files obtained from Illumina sequencing. Sequence reads were aligned to human hg19 reference sequence using Rsubread aligner. The data were normalized using TMM (trimmed mean of M values) method. Differentially expressed cellular genes were identified using edgeR. RNA seq data of GC B cell samples (GSM1129344, GSM1129345, GSM1129346 and GSM1129347) and primary DLBCL (GSE45982) were similarly aligned and normalized. Only genes which had counts-per-million greater than 1 in at least four samples were considered. Criteria used were p value <0.05 and absolute fold change >2.0. Gene ontology (GO) analysis was performed using the DAVID Gene Functional Classification Tool (http://david.abcc.ncifcrf.gov). For the analysis of overlapping gene sets we used a list of collagen genes from: http://www.genenames.org /genefamilies/COLLAGEN.

***Re-analysis of published data***

We re-analyzed data reported by Reddy et al. For each of 624 cases, we defined a total copy number score by summing the number of significant copy number losses or gains among the 140 genes included in the copy number analysis. Cases were split into high and low DDR1 expression (above and below the median) and further subdivided into those with wild-type or mutated TP53 gene. The frequency of SCNA among subgroups were compared using the Mann Whitney U test.
